# The Structural Combination of SIL and MODAG Scaffolds Fails to Enhance Binding to α-Synuclein but Reveals Promising Affinity to Amyloid β

**DOI:** 10.3390/molecules28104001

**Published:** 2023-05-10

**Authors:** Adriana Di Nanni, Ran Sing Saw, Gregory D. Bowden, Natasha S. R. Bidesi, Kaare Bjerregaard-Andersen, Špela Korat, Matthias M. Herth, Bernd J. Pichler, Kristina Herfert, Andreas Maurer

**Affiliations:** 1 Werner Siemens Imaging Center, Department of Preclinical Imaging and Radiopharmacy, Eberhard Karls University Tübingen, 72076 Tübingen, Germany; 2 Cluster of Excellence iFIT (EXC 2180) “Image-Guided and Functionally Instructed Tumor Therapies”, Eberhard Karls University Tübingen, 72076 Tübingen, Germany; 3 Department of Drug Design and Pharmacology, Faculty of Health and Medicinal Sciences, University of Copenhagen, Jagtvej 160, 2100 Copenhagen, Denmark; 4 Department of Antibody Engineering and Biochemistry, H. Lundbeck A/S, Ottiliavej 9, 2500 Copenhagen, Denmark; 5 Department of Radiology and Nuclear Medicine, Amsterdam UMC, Vrije Universiteit Amsterdam, De Boelelaan, 1117 Amsterdam, The Netherlands; 6 Amsterdam Neuroscience, Brain Imaging, 1117 Amsterdam, The Netherlands; 7 Department of Clinical Physiology, Nuclear Medicine & PET, Rigshospitalet, Blegdamsvej 9, 2100 Copenhagen, Denmark

**Keywords:** α-synuclein, positron emission tomography, PET tracer, phenothiazine, 3,5-diphenylpyrazole, PD, MSA, DLB

## Abstract

A technique to image α-synuclein (αSYN) fibrils in vivo is an unmet scientific and clinical need that would represent a transformative tool in the understanding, diagnosis, and treatment of various neurodegenerative diseases. Several classes of compounds have shown promising results as potential PET tracers, but no candidate has yet exhibited the affinity and selectivity required to reach clinical application. We hypothesized that the application of the rational drug design technique of molecular hybridization to two promising lead scaffolds could enhance the binding to αSYN up to the fulfillment of those requirements. By combining the structures of SIL and MODAG tracers, we developed a library of diarylpyrazoles (DAPs). In vitro evaluation through competition assays against [^3^H]SIL26 and [^3^H]MODAG−001 showed the novel hybrid scaffold to have preferential binding affinity for amyloid β (Aβ) over αSYN fibrils. A ring-opening modification on the phenothiazine building block to produce analogs with increased three-dimensional flexibility did not result in an improved αSYN binding but a complete loss of competition, as well as a significant reduction in Aβ affinity. The combination of the phenothiazine and the 3,5-diphenylpyrazole scaffolds into DAP hybrids did not generate an enhanced αSYN PET tracer lead compound. Instead, these efforts identified a scaffold for promising Aβ ligands that may be relevant to the treatment and monitoring of Alzheimer’s disease (AD).

## 1. Introduction

Neurodegenerative diseases such as Parkinson’s disease (PD), multiple system atrophy (MSA), and dementia with Lewy bodies (DLB) share the accumulation of α-synuclein (αSYN) fibrils as a hallmark of pathogenesis [1,2]. Although a cell-to-cell prion-like spreading of αSYN oligomers has been widely suggested [3,4], the protein’s role in disease progression is still not fully understood [5,6]. A technique to image αSYN fibrils in vivo would be invaluable to the advancement of the field, providing a tool for the early diagnosis and scientific study of synucleinopathies as well as the evaluation of new potential therapies.

Positron emission tomography (PET) is a non-invasive technique whose sensitivity allows the imaging of protein aggregates in vivo. This approach is already clinically established for the quantitative detection of amyloid β (Aβ) plaques in Alzheimer’s disease (AD) patients [7]. However, αSYN fibrils are much less abundant (at least 10-fold lower density) [8]; they are located intracellularly and are often accompanied by age-related structurally similar Aβ and tau fibrils. These factors demand molecular probes with exceptionally high affinities and selectivities for the target, making the implementation of an in vivo imaging approach to synucleinopathies significantly more challenging. Despite the development of multiple promising compound classes [9], no αSYN PET tracer is currently validated for clinical use.

**Figure 1:**
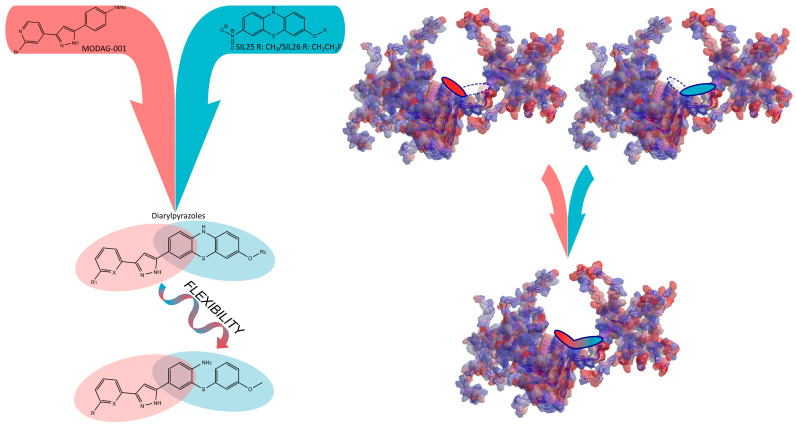
The development of DAP hybrid compounds by the combination of the promising αSYN ligands MODAG−001 and SIL5/SIL26 as an attempt to achieve interaction with a larger portion of the binding site. αSYN structure described by solid-state NMR [24] (PDB ID: 2N0A) was visualized by Chem3D 20.1 (PerkinElmer Informatics, Waltham, MA, USA) and the ligands’ interaction with the binding site 3/13 [19] was schematically illustrated.

Phenothiazines stand out as promising structures among the αSYN ligands evaluated so far. Yu et al. developed a library of phenothiazine-based analogs, and the in vitro assessment of their binding properties via fibril assays led to the identification of SIL5 and SIL26 (Figure 1), with encouraging affinities to αSYN for both (*K*_i-SIL5_ = 66.2 nM, *K*_i-SIL26_ = 15.5 nM) and moderate selectivity over Aβ (*K*_i-SIL26_ = 103 nM) and tau (*K*_i-SIL26_ = 125 nM) for the latter [10,11,12].

In addition to these efforts, Wagner et al. performed a systematic high-throughput screening combined with structure–activity relationship (SAR) studies to identify anle138b from a library of 3,5-diphenylpyrazoles as an inhibitor of αSYN fibril aggregation, highlighting the compound’s specific binding to pathological αSYN oligomers [13]. In parallel to its phase 1 clinical trial evaluation as a potential therapeutic agent [14,15,16], the lead compound was further developed into a series of novel carbon-11 labeled PET tracers, such as [^11^C]anle253b (IC_50_ = 1.6 nM) and [^11^C]MODAG−001 (*K*_d_ = 0.6 ± 0.1 nM) [17,18].

Recent in silico studies revealed the existence of multiple binding sites in αSYN fibrils, with different classes of compounds interacting with distinct portions of the fibrils: Y39-S42-T44 (site 2), G86-F94-K96 (site 9), and K43-K45-V48-H50 (site 3/13) were the clefts identified as putative binding sites. SIL26 showed preferential binding to the positively charged site 3/13, while anle138b was not included in the investigation [19]. As the two lead compounds exhibited reciprocal displacement in competition binding assays [18], we hypothesize that they interact with overlapping sections of the same site. A scaffold accommodating a larger portion of the site (Figure 1) would produce a greater release of free energy upon binding, resulting in higher affinity.

The combination of two promising scaffolds is a widespread approach in medicinal chemistry. Numerous ligands have been conjugated by a linker, fused, or merged to improve their properties (e.g., higher affinity and selectivity, enhanced pharmacokinetics and pharmacodynamics, reduced toxicity) [20,21] or integrate their activity towards different targets [22,23]. By applying molecular hybridization as the drug design technique, this study aims at merging two established αSYN scaffolds (phenothiazine and 3,5-diphenylpyrazole) in an attempt to produce a novel ligand with improved binding properties suitable for the development of an αSYN PET tracer.

## 2. Results and Discussion

### 2.1. Development of a DAP Library by the Combination of SIL and MODAG Scaffolds

**Scheme 1:**
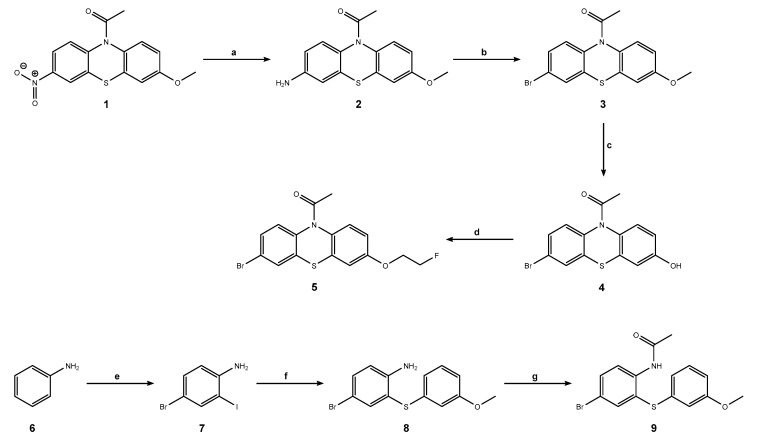
Synthesis of phenothiazine-based building blocks: (**a**) NiCl_2_∙6H_2_O, NaBH_4_, MeCN/H_2_O, r.t., 15 min; (**b**) CuBr_2_, *t*-BuONO, MeCN, 0 °C to r.t., 3 h; (**c**) BBr_3_ 1M in DCM, DCM, −78 °C to r.t., overnight; (**d**) NaH, 1-bromo-2-fluoroethane, DMF, 0 °C to r.t., overnight; (**e**) NH_4_OAc 0.1% in MeOH, NBS, NIS, MeCN, r.t., 45 min; (**f**) 3-methoxythiophenol, K_2_CO_3_, NMP, 100 °C, overnight; (**g**) pyridine, acetic anhydride, r.t., 3 h.

**Scheme 2:**
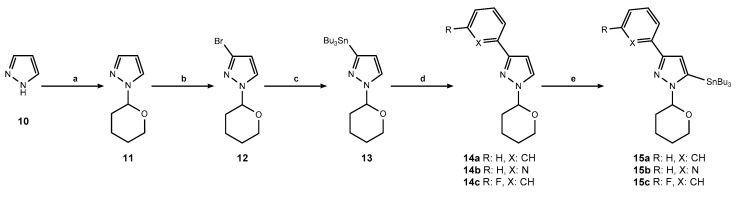
Synthesis of pyrazole-based building blocks: (**a**) DHP, TFA, reflux, 45 min; (**b**) *n*-BuLi, Br_2_, THF, −78 °C, 1 h; (**c**) (SnBu_3_)_2_, Pd(PPh_3_)_4_, toluene, 100 °C, overnight; (**d**) aryl bromide, Pd(PPh_3_)_4_, NMP, 100 °C, overnight; (**e**) *n*-BuLi, SnBuCl_3_, THF, −78 °C, 1 h.

**Scheme 3:**
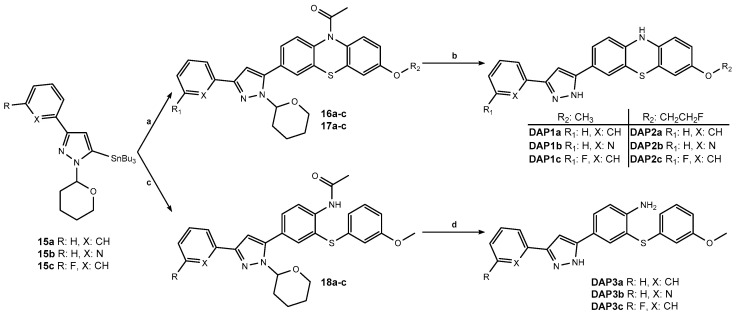
Synthesis of DAP hybrid compounds: (**a**) **3** or **5**, Pd(PPh_3_)_4_, NMP, 90 °C, overnight; (**b**) HCl 37%, MeOH/H_2_O, 80 °C, 6 h; (**c**) **9**, Pd(PPh_3_)_4_, DMF, 110 °C, overnight; (**d**) HCl 37%, MeOH/H_2_O, 80 °C, 6 h.

Two sets of diarylpyrazole (DAP) hybrid compounds were designed and developed based on the combination of the parent scaffolds (Figure 1) to produce a new class of compounds with promising αSYN binding. Bromo-substituted SIL5 (**3**) and SIL26 (**5**) analogs were synthesized by the NiCl_2_-catalyzed reduction of the nitro group into a primary amine followed by bromination, using *t*-BuONO and CuBr_2_ (Scheme 1) [25,26]. The 3-bromo phenothiazines were reacted in Pd(PPh_3_)_4_-catalyzed cross-couplings with diversely substituted THP-protected 3-aryl stannylpyrazoles followed by acidic deprotection to produce a small library of novel derivatives (Scheme 2 and Scheme 3). Their binding properties were evaluated through competition binding assays against [^3^H]SIL26 and [^3^H]MODAG−001 on human recombinant αSYN fibrils and synthetic Aβ_1-42_ fibrils. The in vitro results reported in Table 1 show that DAP hybrids have a significantly lower affinity towards αSYN than both parent scaffolds, with a minimum of 6-fold reduction compared to SIL26 and a 117- to 848-fold decrease compared to MODAG−001 [11,18]. These findings disprove our hypothesis of enhanced binding properties resulting from the combination of pharmacophores from SIL and MODAG structures.

SAR studies carried out by Wagner et al. on the first set of 3,5-diphenylpyrazoles demonstrated a loss of activity when the bromine substitution is transferred from the *meta* to the *ortho* position, highlighting the importance of a planar ligand conformation [13]. We speculate that the constrained flat structure of phenothiazines hampers a successful interaction with the binding site by interrupting the linearity of the two phenyl rings bound to the pyrazole.

**Figure 2:**
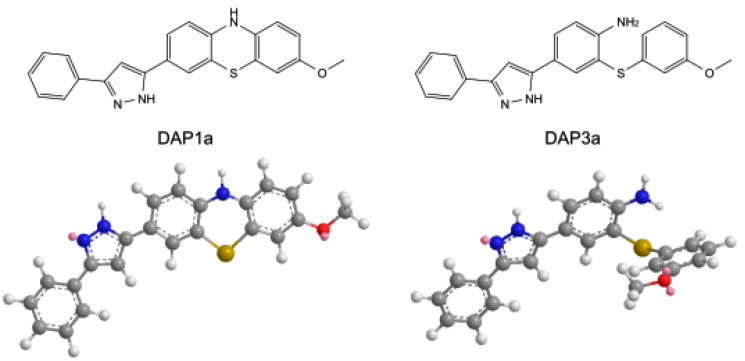
Predicted 3D structure of **DAP1a** and **DAP3a** (MM2 energy minimization calculated by Chem3D 20.1, PerkinElmer Informatics, Waltham, MA, USA).

Interestingly, the binding affinity of DAP hybrids to Aβ fibrils was in the range of 1 to 10 nM for most analogs (Table 1), 1 to 2 orders of magnitude lower than the parent scaffold SIL26. Their low *K*_i_ values alone are not sufficient to make our hybrid compounds attractive as potential Aβ PET tracers as they are comparable to the FDA-approved compounds (Florbetaben: *K*_i_ = 6.70 ± 0.30 nM, Florbetapir: *K*_d_ = 3.72 ± 0.30 nM, Flutemetamol: *K*_i_ = 0.74 ± 0.38 nM) [27,28]. An Aβ-focused assay comparing DAPs with the abovementioned tracers would be required to validate the correlation. However, our hybrid compounds represent a novel scaffold whose theranostic profile is worth further investigation. In fact, a library of rhodanine-substituted phenothiazines developed by Dao et al. exhibited inhibition of Aβ_1-42_ aggregation with IC_50_ values as low as 0.67 ± 0.02 µM and also induced the disaggregation of preformed Aβ_1-42_ fibrils (IC_50_ = 0.82 ± 0.10 µM) [29]. As DAP’s overall structure and predicted conformation (Figure 2) partially overlap with these fluorescent probes, we speculated that they could also share some of their physiological properties. An additional study specifically investigating these traits will be necessary in order to confirm our hypothesis.

### 2.2. Enhancement of Structural Flexibility in Ring-Opened DAP Analogs

Based on the hypothesis that the constrained three-dimensional conformation of the phenothiazine ring prevents a successful interaction with the binding site, more flexible DAP hybrid compounds were synthesized to afford a set of analogs that would adjust more effectively to the binding site.

The NH_4_OAc-catalyzed bromination procedure proposed by Das et al. was modified into a one-pot, sequential substitution of aniline with *N*-bromosuccinimide and *N*-iodosuccinimide to generate 2-iodo-4-bromoaniline (**7**) [30]. The intermediate was further reacted with 3-methoxythiophenol, followed by *N*-acetylation, to produce **9**, the ring-opened analog of the phenothiazine-based compound **3** (Scheme 1). As in the previously established synthetic pathway, Pd(PPh_3_)_4_-catalyzed cross-coupling and acidic deprotection afforded a selection of ring-opened DAP hybrid compounds (Scheme 3).

The minor structural alteration significantly changed the molecule’s three-dimensional architecture, conferring more conformational freedom and potentially favoring the binding affinity. Energy minimization of **DAP1a** and **DAP3a** structures revealed a discontinuance of the planarity in the diphenyl thioether moiety of the **DAP3a** most stable conformation (Figure 2). Together with an electronic change arising from the replacement of a heteroaromatic amine with an aniline, this transformation led to the formation of molecules with very different properties. However, in in vitro assays, all compounds showed a complete loss of competition for αSYN fibrils when tested against both [^3^H]SIL26 and [^3^H]MODAG−001 (Table 2), invalidating our hypothesis on flexibility. Additionally, a significant decrease in affinity to Aβ was also observed, highlighting the criticality of three-dimensional conformation in designing new ligands to differentiate between misfolded proteins.

### 2.3. Interaction with αSYN Binding Sites

**Figure 3:**
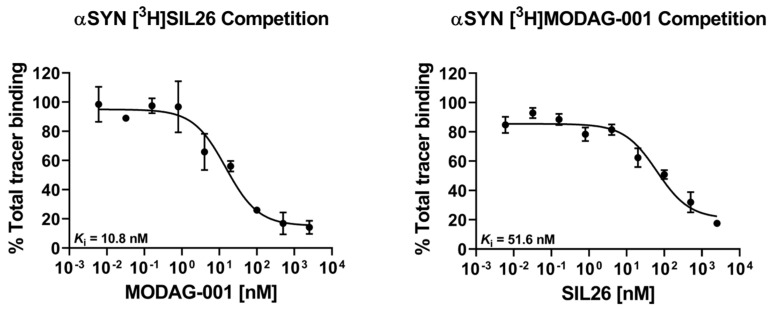
Competition between SIL26 and MODAG−001 for the binding to αSYN fibrils (three separate experiments per curve).

Our findings demonstrated that the parent scaffolds compete with each other for the binding to αSYN as SIL26 displaces [^3^H]MODAG−001 with a *K*_i_ of 51.6 nM and MODAG−001 displaces [^3^H]SIL26 with a *K*_i_ of 10.8 nM (Figure 3). These results agreed with the previous literature [18] and aligned with the higher affinity expected for the 3,5-diphenylpyrazole, leading to the hypothesis that the two classes share the same interaction site.

The biological evaluation of the DAP hybrid compounds did not support our hypothesis. We speculate that the competition between the parent scaffolds results from the existence of two distinct binding sites partially overlapping with each other. Therefore, to successfully fit the interaction site, a different molecular hybridization approach may be necessary, e.g., the introduction of a linker connecting the two moieties or a greater overlap of the parent scaffolds to generate a smaller compound.

A recent solid-state nuclear magnetic resonance spectroscopy (ssNMR) study describes the most stable binding mode of anle138b to αSYN as the end-to-end filling of a tubular cavity [31]. This model raises concerns regarding the overall size of the DAP hybrids, which might partially obstruct their interaction with the fibrils, producing an additional cause for low binding affinity.

## 3. Materials and Methods

### 3.1. Chemistry

All chemicals were purchased from Sigma Aldrich (St. Louis, Missouri, USA), abcr GmbH (Karlsruhe, Germany), or Carl Roth (Karlsruhe, Germany) and used without any further purification.

Reaction progress was monitored by thin-layer chromatography (TLC) on 0.20 mm Polygram SIL G/UV_254_ (silica gel 60) TLC plates (Macherey-Nagel, Düren, Germany) with the chosen eluent mixture and/or analytical HPLC-MS (ESI detector, Agilent, Santa Clara, CA, USA) equipped with a Luna 5 µm C18 (2) 100 Å 50 × 2 mm column (Phenomenex, Torrance, California, USA) under the following gradient: 0–7.60 min (0% to 100% B), 7.60–8.80 (100% B), 8.80–9.30 min (100% to 0% B), 9.30–13.0 min (0% B); solvent A: 0.1% formic acid in H_2_O; solvent B: MeCN; 0.4 mL/min.

Purification was performed through automated flash chromatography on an Isolera 4 system (Biotage, Uppsala, Sweden).

^1^H and ^13^C NMR spectra were acquired on an Avance III AV 600 (^1^H: 600.13 MHz; ^13^C: 150.61 MHz) spectrometer (Bruker, Billerica, MA, USA). All chemical shifts (δ) are reported as parts per million (ppm) and referenced to residual solvent peaks (DMSO-d_6_: δ_H_ = 2.50, δ_C_ = 39.52).

HPLC-MS chromatograms and ^1^H NMR spectra of compounds **DAP1a-3c** are reported in the Supplementary Information.

#### 3.1.1. General Procedure A (Compounds **14a**–**14c**)

To a solution of **13** (1.13 mmol) and the selected aryl bromide (2.27 mmol) in NMP (3.50 mL) under argon atmosphere was added Pd(PPh_3_)_4_ (5% mol). The mixture was stirred overnight at 100 °C. The crude product mixture was diluted in water and extracted with EtOAc. The organic phase was dried over MgSO_4_, evaporated under reduced pressure, and purified by flash chromatography (PE/EtOAc).

#### 3.1.2. General Procedure B (Compounds **15a**–**15c**)

A solution of the selected 3-aryl-1-(tetrahydro-2*H*-pyran-2-yl)-pyrazole (0.63 mmol) in THF (2.50 mL) was cooled to −78 °C under a positive pressure of argon. A solution of *n*-BuLi 2.5M in hexane (0.76 mmol) was added dropwise, after which the mixture was stirred for 15 min. SnBu_3_Cl (0.76 mmol) was added dropwise, and the resulting mixture was stirred for 1 h, allowing it to reach room temperature. The reaction mixture was poured into water and extracted with EtOAc. The organic phase was dried over MgSO_4_, evaporated under reduced pressure, and purified by flash chromatography (PE/EtOAc).

#### 3.1.3. General Procedure C (Compounds **16a**–**17c**)

To a solution of the selected 3-aryl-1-(tetrahydro-2*H*-pyran-2-yl)-5-(tributylstannyl)-pyrazole (0.56 mmol) and the selected 3-bromo-7-OR_2_-*N*-acetylphenothiazine (0.73 mmol) in NMP (8.50 mL) was added Pd(PPh_3_)_4_ (5% mol) under an argon atmosphere. The mixture was stirred overnight at 90 °C. The reaction mixture was diluted with water and extracted with EtOAc. The organic phase was dried over MgSO_4_, evaporated under reduced pressure, and purified by flash chromatography (PE/EtOAc).

#### 3.1.4. General Procedure D (Compounds **18a**–**18c**)

To a solution of the selected 3-aryl-1-(tetrahydro-2*H*-pyran-2-yl)-5-(tributylstannyl)-pyrazole (0.31 mmol) and **9** (0.40 mmol) in DMF (5.00 mL) was added Pd(PPh_3_)_4_ (10% mol), under an argon atmosphere. The mixture was stirred overnight at 110 °C. The crude was diluted with water and extracted with EtOAc. The organic phase was dried over MgSO_4_, evaporated under reduced pressure, and purified by flash chromatography (PE/EtOAc).

#### 3.1.5. General Procedure E (DAP1a-DAP3c)

To a suspension of the selected protected DAP compound (0.26 mmol) in MeOH/H_2_O 1:1 *v*/*v* (13.0 mL, 13.0 mL), HCl 37% (1.30 mL) was added, and the reaction was stirred at 80 °C for 6 h. The mixture was poured into water, neutralized with NaOH aq. 18 M, and extracted with EtOAc. The organic phase was dried over MgSO_4_, evaporated under reduced pressure, and purified by flash chromatography (PE/EtOAc or DCM/MeOH).

#### 3.1.6. 1-(3-Methoxy-7-nitro-10*H*-phenothiazin-10-yl)ethan-1-one (**1**)

The three-step synthesis was carried out according to the literature procedure [10], starting from 2-amino-6-methoxybenzo[d]thiazole and 1-chloro-2,4-dinitrobenzene. The product was afforded as an orange solid (13.1 g, 58%), with all analytical data corresponding to the published data. R*_f_*: 0.43 (PE/EtOAc 1:1). ^1^H NMR (600 MHz, DMSO-*d_6_*) δ 8.39 (d, *J* = 2.6 Hz, 1H), 8.21 (dd, *J* = 8.8, 2.6 Hz, 1H), 7.82 (d, *J* = 8.8 Hz, 1H), 7.58 (d, *J* = 8.8 Hz, 1H), 7.17 (d, *J* = 2.8 Hz, 1H), 6.99 (dd, *J* = 8.8, 2.8 Hz, 1H), 3.79 (s, 3H), 2.16 (s, 3H). ^13^C NMR (151 MHz, DMSO-*d_6_*) δ 168.6, 158.0, 145.3, 144.5, 133.7, 132.2, 130.4, 128.2, 128.0, 122.8, 122.3, 114.1, 112.5, 55.7, 22.7. HPLC-MS (ESI): *m*/*z* calcd for C_15_H_12_N_2_O_4_S 316.05; [M + H]^+^ found 317.10.

#### 3.1.7. 1-(3-Amino-7-methoxy-10*H*-phenothiazin-10-yl)ethan-1-one (**2**)

To a solution of **1** (13.1 g, 41.4 mmol) in MeCN/H_2_O 10:1 *v*/*v* (260 mL, 26.0 mL) was added NiCl_2_∙6H_2_O (1.97 g, 8.28 mmol), and the mixture was stirred at room temperature for 5 min. NaBH_4_ (6.26 g, 165 mmol) was then added in portions, and the reaction was stirred at room temperature for a further 15 min. The reaction mixture was diluted with water and extracted with DCM. The organic phase was washed with water, dried over MgSO_4_, and evaporated to afford the product as a brown solid (11.0 g, 93%). R*_f_*: 0.20 (PE/EtOAc 1:1). ^1^H NMR (600 MHz, DMSO-*d_6_*) δ 7.42 (d, *J* = 8.8 Hz, 1H), 7.19 (d, *J* = 8.5 Hz, 1H), 7.05 (d, *J* = 2.8 Hz, 1H), 6.89 (dd, *J* = 8.8, 2.8 Hz, 1H), 6.66 (d, *J* = 2.4 Hz, 1H), 6.53 (dd, *J* = 8.5, 2.4 Hz, 1H), 5.30 (s, 2H), 3.76 (s, 3H), 2.04 (s, 3H). ^13^C NMR (151 MHz, DMSO-*d_6_*) δ 168.9, 157.2, 147.4, 132.4, 127.8, 127.8, 127.4, 127.3, 127.3, 112.8, 112.5, 112.0, 111.4, 55.5, 22.4. HPLC-MS (ESI): *m*/*z* calcd for C_15_H_14_N_2_O_2_S 286.08; [M + H]^+^ found 287.10.

#### 3.1.8. 1-(3-Bromo-7-methoxy-10*H*-phenothiazin-10-yl)ethan-1-one (**3**)

A solution of **2** (3.00 g, 10.5 mmol) in MeCN (1.00 L) was cooled to 0 °C under an argon atmosphere, and CuBr_2_ (3.51 g, 15.7 mmol) was added. *t*-BuONO (2.08 mL, 15.7 mmol) was added dropwise at 0 °C, and the mixture was stirred at room temperature for 3 h. The reaction was quenched with H_3_NSO_3_ aq. 48.5M (1.00 L) and extracted four times with EtOAc. The organic phase was dried over MgSO_4_, evaporated under reduced pressure, and purified by flash chromatography (PE/EtOAc 5% to 45% B) to afford the product as a pink solid (1.07 g, 29%). R*_f_*: 0.51 (PE/EtOAc 1:1). ^1^H NMR (600 MHz, DMSO-*d_6_*) δ 7.79 (d, *J* = 2.2 Hz, 1H), 7.56 (dd, *J* = 8.5, 2.2 Hz, 1H), 7.52 (d, *J* = 8.5 Hz, 2H), 7.13 (d, *J* = 2.8 Hz, 1H), 6.96 (dd, *J* = 8.8, 2.8 Hz, 1H), 3.78 (s, 3H), 2.10 (s, 3H). ^13^C NMR (151 MHz, DMSO-*d_6_*) δ 168.6, 157.7, 138.3, 138.3, 134.4, 131.2, 129.9, 129.9, 129.0, 128.0, 119.0, 113.7, 112.4, 55.7, 22.6. HPLC-MS (ESI): *m*/*z* calcd for C_15_H_12_BrNO_2_S 348.98; [M + H]^+^ found 349.95, 351.90.

#### 3.1.9. 1-(3-Bromo-7-hydroxy-10*H*-phenothiazin-10-yl)ethan-1-one (**4**)

A solution of **3** (1.05 g, 3.01 mmol) in DCM (45.0 mL) was cooled to −78 °C under an argon atmosphere, and BBr_3_ 1M in DCM (10.5 mL) was added. The reaction was stirred overnight at room temperature. The crude was poured into water and extracted with DCM. The organic phase was dried over MgSO_4_, evaporated, and further purified by flash chromatography (PE/EtOAc 8% to 66% B) to afford the product as a pink solid (632 mg, 63%). R*_f_*: 0.37 (PE/EtOAc 1:1). ^1^H NMR (600 MHz, DMSO-*d_6_*) δ 9.90 (s, 1H), 7.77 (dd, *J* = 2.1, 1.1 Hz, 1H), 7.54 (dd, *J* = 8.3, 2.1 Hz, 1H), 7.50 (t, *J* = 8.3 Hz, 1H), 7.40 (d, *J* = 8.7 Hz, 1H), 6.89 (d, *J* = 2.7 Hz, 1H), 6.78 (dd, *J* = 8.7, 2.7 Hz, 1H), 2.09 (s, 3H). ^13^C NMR (151 MHz, DMSO-*d_6_*) δ 168.7, 156.1, 138.5, 138.4, 134.5, 132.4, 129.8, 129.8, 129.1, 128.0, 118.9, 114.6, 113.8, 22.6. HPLC-MS (ESI): *m*/*z* calcd for C_14_H_10_BrNO_2_S 334.96; [M + H]^+^ found 336.15, 337.95.

#### 3.1.10. 1-(3-Bromo-7-(2-fluoroethoxy)-10*H*-phenothiazin-10-yl)ethan-1-one (**5**)

A solution of **4** (365 mg, 0.98 mmol) in dry DMF (24.0 mL) was cooled to 0 °C under an argon atmosphere, and a NaH 60% dispersion in mineral oil (58.6 mg, 1.47 mmol) was added. The mixture was stirred at 0 °C for 15 min. 1-Bromo-2-fluoroethane (0.11 mL, 1.47 mmol) was added dropwise, and the reaction was stirred overnight at room temperature. The reaction mixture was poured into water (220 mL) and extracted with EtOAc. The organic phase was dried over MgSO_4_, evaporated under reduced pressure, and purified by flash chromatography (PE/EtOAc 8% to 66% B) to afford the product as a pink solid (301 mg, 91%). R*_f_*: 0.18 (PE/EtOAc 2:1). ^1^H NMR (600 MHz, DMSO-*d_6_*) δ 7.79 (d, *J* = 2.1 Hz, 1H), 7.56 (dd, *J* = 8.4, 2.1 Hz, 1H), 7.55–7.47 (m, 2H), 7.18 (d, *J* = 2.8 Hz, 1H), 7.00 (dd, *J* = 8.8, 2.8 Hz, 1H), 4.73 (dt, *J* = 47.9, 3.9 Hz, 2H), 4.27 (dq, *J* = 30.4, 4.4 Hz, 2H), 2.10 (s, 3H). ^13^C NMR (151 MHz, DMSO-*d_6_*) δ 168.6, 156.6, 138.3, 138.3, 131.5, 130.0, 130.0, 129.9, 129.0, 128.1, 119.0, 114.2, 113.0, 82.0 (d, *J* = 166.6 Hz), 67.6 (d, *J* = 18.9 Hz), 22.6. HPLC-MS (ESI): *m*/*z* calcd for C_16_H_13_BrFNO_2_S 380.98; [M + H]^+^ found 382.05, 384.00.

#### 3.1.11. 4-Bromo-2-iodoaniline (**7**)

NH_4_OAc 0.1% in MeOH (1.50 mL) was added to a solution of aniline (1.50 mL, 16.4 mmol) in MeCN (75.0 mL). *N*-Bromosuccinimide (2.92 g, 16.4 mmol) was added in portions, and the mixture was stirred at room temperature for 15 min. HPLC-MS confirmed that the mono-substitution of the aniline was achieved. *N*-Iodosuccinimide (4.07 g, 18.1 mmol) was added in portions, and the mixture was stirred at room temperature for 30 min. The solvent was concentrated under a vacuum and the crude was poured into water and extracted with EtOAc. The organic phase was dried over MgSO_4_, evaporated under reduced pressure, and purified by flash chromatography (PE/EtOAc 10% to 20% B) to afford the product (1.98 g, 40%). R*_f_*: 0.64 (PE/EtOAc 1:1). ^1^H NMR (600 MHz, DMSO-*d_6_*) δ 7.65 (d, *J* = 2.3 Hz, 1H), 7.21 (dd, *J* = 8.6, 2.3 Hz, 1H), 6.70 (d, *J* = 8.6 Hz, 1H), 5.37 (s, 2H). ^13^C NMR (151 MHz, DMSO-*d_6_*) δ 148.1, 139.3, 131.5, 115.5, 106.6, 83.4. HPLC-MS (ESI): *m*/*z* calcd for C_6_H_5_BrIN 296.87; found [M + H]^+^ 297.90, 299.85.

#### 3.1.12. 4-Bromo-2-((3-methoxyphenyl)thio)aniline (**8**)

3-Methoxythiophenol (0.81 mL, 6.54 mmol) and potassium carbonate (1.09 g, 7.85 mmol) were added to a solution of **7** (1.95 g, 6.54 mmol) in NMP (20.0 mL) under argon atmosphere. CuI (62.3 mg, 5% mol) was added, and the mixture was stirred overnight at 100 °C. The reaction mixture was poured into water and extracted with EtOAc. The organic phase was dried over MgSO_4_, evaporated under reduced pressure, and purified by flash chromatography (PE/EtOAc 1% to 15% B) to afford the product (748 mg, 37%). R*_f_*: 0.44 (PE/EtOAc 5:1). ^1^H NMR (600 MHz, DMSO-*d_6_*) δ 7.42 (d, *J* = 2.4 Hz, 1H), 7.30 (dd, *J* = 8.6, 2.4 Hz, 1H), 7.20 (t, *J* = 8.3 Hz, 1H), 6.78 (d, *J* = 8.6 Hz, 1H), 6.75 (ddd, *J* = 8.3, 2.4, 1.0 Hz, 1H), 6.66–6.62 (m, 2H), 5.59 (s, 2H), 3.69 (s, 3H). ^13^C NMR (151 MHz, DMSO-*d_6_*) δ 159.7, 149.6, 137.9, 137.1, 133.5, 130.1, 119.0, 116.7, 114.1, 112.6, 111.2, 105.9, 55.1. HPLC-MS (ESI): *m*/*z* calcd for C_13_H_12_BrNOS 308.98; [M + H]^+^ found 309.90, 312.00.

#### 3.1.13. *N*-(4-Bromo-2-((3-methoxyphenyl)thio)phenyl)acetamide (**9**)

Pyridine (3.5 mL) was added to a solution of **8** (735 mg, 2.37 mmol) in acetic anhydride (20.0 mL), and the mixture was stirred at room temperature for 3 h. The reaction mixture was poured into water and extracted with EtOAc. The organic phase was dried over MgSO_4_, evaporated under reduced pressure, and purified by flash chromatography (PE/EtOAc 1% to 20% B) to afford the product (708 mg, 85%). R*_f_*: 0.28 (PE/EtOAc 5:1). ^1^H NMR (600 MHz, DMSO-*d_6_*) δ 9.43 (d, *J* = 22.9 Hz, 1H), 7.56 (d, *J* = 8.6 Hz, 1H), 7.49 (dd, *J* = 8.6, 2.4 Hz, 1H), 7.35 (d, *J* = 2.4 Hz, 1H), 7.31 (t, *J* = 8.2 Hz, 1H), 6.94–6.89 (m, 1H), 6.85 (d, *J* = 8.6 Hz, 1H), 6.85 (s, 1H), 3.73 (s, 3H), 2.00 (s, 3H). ^13^C NMR (151 MHz, DMSO-*d_6_*) δ 168.6, 159.8, 137.0, 134.8, 133.9, 130.9, 130.9, 130.5, 127.3, 122.8, 117.3, 116.1, 113.5, 55.2, 23.1. HPLC-MS (ESI): *m*/*z* calcd for C_15_H_14_BrNO_2_S 350.99; [M + H]^+^ found 352.05, 354.00.

#### 3.1.14. 1-(Tetrahydro-2*H*-pyran-2-yl)-pyrazole (**11**)

To a solution of 1*H*-pyrazole (1.40 g, 21.0 mmol) in 3,4-dihydro-2*H*-pyran (2.57 mL, 27.3 mmol), TFA (0.50 mL, 6.21 mmol) was added. The mixture was refluxed for 45 min. The reaction mixture was diluted in water and extracted with EtOAc. The organic phase was dried over MgSO_4_, evaporated under reduced pressure, and purified by flash chromatography (PE/EtOAc 8% to 65% B) to afford the product as a colorless oil (2.97 g, 93%). R*_f_*: 0.48 (PE/EtOAc 2:1). ^1^H NMR (600 MHz, DMSO-*d_6_*) δ 7.84 (dd, *J* = 2.4, 0.7 Hz, 1H), 7.47 (dd, *J* = 1.7, 0.7 Hz, 1H), 6.29 (dd, *J* = 2.4, 1.7 Hz, 1H), 5.40 (dd, *J* = 10.0, 2.5 Hz, 1H), 3.91 (dtd, *J* = 11.5, 3.8, 1.9 Hz, 1H), 3.67–3.57 (m, 1H), 2.15–2.02 (m, 1H), 1.97–1.86 (m, 2H), 1.71–1.62 (m, 1H), 1.57–1.50 (m, 2H). ^13^C NMR (151 MHz, DMSO-*d_6_*) δ 138.7, 128.6, 105.7, 86.7, 66.8, 30.0, 24.7, 22.1. HPLC-MS (ESI): *m*/*z* calcd for C_8_H_12_N_2_O 152.09; [M + H]^+^ found 153.15.

#### 3.1.15. 3-Bromo-1-(tetrahydro-2*H*-pyran-2-yl)-pyrazole (**12**)

A solution of **11** (2.97 g, 152 mmol) in THF (22.0 mL) was cooled to −78 °C under argon atmosphere. A solution of *n*-BuLi 2.5M in hexane (10.1 mL, 25.3 mmol) was added dropwise. Bromine (1.30 mL, 25.3 mmol) was added dropwise, and the mixture was stirred for 1 h. The reaction mixture was poured into water and extracted with EtOAc. The organic phase was dried over MgSO_4_, evaporated under reduced pressure, and heated at 150 °C for a few seconds to allow conversion into the thermodynamically more stable 3-bromo regioisomer. It was purified by flash chromatography (PE/EtOAc 6% to 20% B) to afford the product as a light yellow oil (1.84 g, 41%). R*_f_*: 0.33 (PE/EtOAc 2:1). ^1^H NMR (600 MHz, DMSO-*d_6_*) δ 7.89 (d, *J* = 2.5 Hz, 1H), 6.43 (d, *J* = 2.4 Hz, 1H), 5.37 (dd, *J* = 9.9, 2.4 Hz, 1H), 3.91 (dtd, *J* = 11.5, 4.0, 1.8 Hz, 1H), 3.66–3.56 (m, 1H), 2.10–2.00 (m, 1H), 1.96–1.85 (m, 2H), 1.70–1.59 (m, 1H), 1.56–1.49 (m, 2H). ^13^C NMR (151 MHz, DMSO-*d_6_*) δ 131.8, 125.0, 108.5, 86.8, 66.8, 29.4, 24.5, 21.8. HPLC-MS (ESI): *m*/*z* calcd for C_3_H_3_BrN_2_ (THP cleaved by acidic conditions) 145.95; [M + H]^+^ found 147.00, 148.95.

#### 3.1.16. 1-(Tetrahydro-2*H*-pyran-2-yl)-3-(tributylstannyl)-pyrazole (**13**)

To a solution of **12** (500 mg, 2.16 mmol) in toluene (6.00 mL), bis(tributyltin) (1.31 mL, 2.60 mmol) and Pd(PPh_3_)_4_ (250 mg, 0.22 mmol) were added under an argon atmosphere. The mixture was stirred overnight at 100 °C. The reaction mixture was diluted in water and extracted with EtOAc. The organic phase was dried over MgSO_4_, evaporated under reduced pressure, and purified by flash chromatography (PE/EtOAc 6% to 12% B) to afford the product as a colorless oil (359 mg, 38%). R*_f_*: 0.46 (PE/EtOAc 9:1). ^1^H NMR (600 MHz, DMSO-*d_6_*) δ 7.86 (d, *J* = 2.3 Hz, 1H), 6.32 (d, *J* = 2.2 Hz, 1H), 5.45 (dd, *J* = 9.9, 2.6 Hz, 1H), 3.90 (dtd, *J* = 11.5, 3.8, 1.8 Hz, 1H), 3.67–3.56 (m, 1H), 2.07 (dddd, *J* = 12.9, 12.1, 9.8, 4.0 Hz, 1H), 1.95 (dtd, *J* = 13.3, 4.0, 1.8 Hz, 1H), 1.89 (dq, *J* = 12.9, 3.6 Hz, 1H), 1.72–1.64 (m, 1H), 1.65–1.55 (m, 2H), 1.55–1.51 (m, 6H), 1.29 (h, *J* = 7.8, 7.2 Hz, 6H), 1.01 (t, *J* = 7.9 Hz, 6H), 0.85 (t, *J* = 7.3 Hz, 9H). ^13^C NMR (151 MHz, DMSO-*d_6_*) δ 150.2, 128.1, 113.7, 86.4, 66.7, 30.1, 28.5, 26.6, 24.7, 22.1, 13.5, 9.4. HPLC-MS (ESI): *m*/*z* calcd for C_20_H_38_N_2_OSn 442.20; [M + H]^+^ found 443.25.

#### 3.1.17. 3-Phenyl-1-(tetrahydro-2*H*-pyran-2-yl)-pyrazole (**14a**)

The synthesis was carried out according to general procedure A (149 mg, 58%). R*_f_*: 0.51 (PE/EtOAc 3:1). ^1^H NMR (600 MHz, DMSO-*d_6_*) δ 7.92 (d, *J* = 2.5 Hz, 1H), 7.81 (dd, *J* = 8.2, 1.3 Hz, 2H), 7.40 (t, *J* = 7.7 Hz, 2H), 7.30 (tt, *J* = 7.4, 1.2 Hz, 1H), 6.77 (d, *J* = 2.4 Hz, 1H), 5.43 (dd, *J* = 10.1, 2.3 Hz, 1H), 3.95 (dtd, *J* = 13.1, 4.1, 1.8 Hz, 1H), 3.69–3.60 (m, 1H), 2.19–2.09 (m, 1H), 1.98–1.90 (m, 2H), 1.74–1.65 (m, 1H), 1.64–1.57 (m, 2H). ^13^C NMR (151 MHz, DMSO-*d_6_*) δ 150.0, 133.2, 130.3, 128.6, 127.6, 125.2, 103.1, 86.9, 66.9, 29.9, 24.6, 22.1. HPLC-MS (ESI): *m*/*z* calcd for C_9_H_8_N_2_ 144.07 and C_14_H_16_N_2_O 228.13; [M + H]^+^ found 145.20, 229.20.

#### 3.1.18. 2-(1-(Tetrahydro-2*H*-pyran-2-yl)-1H-pyrazol-3-yl)pyridine (**14b**)

The synthesis was carried out according to general procedure A (171 mg, 66%). R*_f_*: 0.23 (PE/EtOAc 1:1). ^1^H NMR (600 MHz, DMSO-*d_6_*) δ 8.57 (ddd, *J* = 4.8, 1.8, 1.0 Hz, 1H), 7.96 (t, *J* = 2.4 Hz, 1H), 7.94 (dq, *J* = 7.9, 1.2 Hz, 1H), 7.82 (td, *J* = 7.7, 1.8 Hz, 1H), 7.34–7.28 (m, 1H), 6.86 (t, *J* = 2.2 Hz, 1H), 5.46 (dt, *J* = 10.1, 2.1 Hz, 1H), 3.95 (dtd, *J* = 13.4, 4.2, 1.8 Hz, 1H), 3.69–3.62 (m, 1H), 2.19–2.10 (m, 1H), 1.98–1.92 (m, 2H), 1.74–1.63 (m, 1H), 1.58–1.51 (m, 2H). ^13^C NMR (151 MHz, DMSO-*d_6_*) δ 151.7, 150.9, 149.3, 136.8, 130.5, 122.7, 119.3, 104.4, 87.0, 66.9, 29.8, 24.6, 22.0. HPLC-MS (ESI): *m*/*z* calcd for C_8_H_7_N_3_ 145.06 and C_13_H_15_N_3_O 229.12; [M + H]^+^ found 146.10, 230.10.

#### 3.1.19. 3-(3-Fluorophenyl)-1-(tetrahydro-2*H*-pyran-2-yl)-pyrazole (**14c**)

The synthesis was carried out according to general procedure A (386 mg, 69%). R*_f_*: 0.61 (PE/EtOAc 2:1). ^1^H NMR (600 MHz, DMSO-*d_6_*) δ 7.94 (d, *J* = 2.5 Hz, 1H), 7.66 (dt, *J* = 7.7, 1.2 Hz, 1H), 7.59 (ddd, *J* = 10.6, 2.7, 1.6 Hz, 1H), 7.44 (td, *J* = 8.0, 6.2 Hz, 1H), 7.12 (tdd, *J* = 9.2, 2.7, 0.9 Hz, 1H), 6.83 (d, *J* = 2.5 Hz, 1H), 5.45 (dd, *J* = 10.1, 2.4 Hz, 1H), 3.95 (dtd, *J* = 11.5, 3.8, 1.8 Hz, 1H), 3.69–3.62 (m, 1H), 2.19–2.08 (m, 1H), 2.00–1.92 (m, 2H), 1.75–1.64 (m, 1H), 1.56 (tt, *J* = 7.8, 3.8 Hz, 2H). ^13^C NMR (151 MHz, DMSO-*d_6_*) δ 162.5 (d, *J* = 242.7 Hz), 148.8 (d, *J* = 3.0 Hz), 135.6 (d, *J* = 8.3 Hz), 130.5 (d, *J* = 8.6 Hz), 130.4, 121.1 (d, *J* = 2.8 Hz), 114.1 (d, *J* = 21.2 Hz), 111.6 (d, *J* = 22.4 Hz), 103.5, 86.8, 66.7, 29.7, 24.5, 21.9. HPLC-MS (ESI): *m*/*z* calcd for C_9_H_7_FN_2_ 162.06 and C_14_H_15_FN_2_O 246.12; [M + H]^+^ found 163.05, 247.20.

#### 3.1.20. 3-Phenyl-1-(tetrahydro-2*H*-pyran-2-yl)-5-(tributylstannyl)-pyrazole (**15a**)

The synthesis was carried out according to general procedure B (298 mg, 91%). R*_f_*: 0.74 (PE/EtOAc 5:1). ^1^H NMR (600 MHz, DMSO-*d_6_*) δ 7.80 (dd, *J* = 8.3, 1.2 Hz, 2H), 7.38 (t, *J* = 7.7 Hz, 2H), 7.27 (tt, *J* = 7.4, 1.3 Hz, 1H), 6.74 (t, *J* = 3.9 Hz, 1H), 5.31–5.22 (m, 1H), 3.95 (dtd, *J* = 10.8, 3.6, 1.8 Hz, 1H), 3.68–3.58 (m, 1H), 2.12–2.04 (m, 2H), 2.03–1.95 (m, 1H), 1.72–1.64 (m, 1H), 1.64–1.56 (m, 2H), 1.56–1.47 (m, 6H), 1.31 (h, *J* = 7.3 Hz, 6H), 1.14–1.08 (m, 6H), 0.89–0.85 (m, 9H). ^13^C NMR (151 MHz, DMSO-*d_6_*) δ 149.8, 142.5, 133.5, 128.5, 127.3, 125.2, 112.1, 88.1, 66.9, 28.5, 27.7, 26.6, 26.2, 24.6, 13.5, 10.3. HPLC-MS (ESI): *m*/*z* calcd for C_26_H_42_N_2_OSn 518.23; [M + H]^+^ found 519.00.

#### 3.1.21. 2-(1-(Tetrahydro-2*H*-pyran-2-yl)-5-(tributylstannyl)-1*H*-pyrazol-3-yl)pyridine (**15b**)

The synthesis was carried out according to general procedure B (245 mg, 68%). R*_f_*: 0.25 (PE/EtOAc 5:1). ^1^H NMR (600 MHz, DMSO-*d_6_*) δ 8.56 (ddd, *J* = 4.8, 1.8, 0.9 Hz, 1H), 7.90 (dt, *J* = 8.0, 1.1 Hz, 1H), 7.80 (td, *J* = 7.7, 1.8 Hz, 1H), 7.28 (ddd, *J* = 7.5, 4.8, 1.2 Hz, 1H), 6.88 (t, *J* = 4.0 Hz, 1H), 5.34–5.27 (m, 1H), 3.96 (dtd, *J* = 10.8, 4.2, 1.8 Hz, 1H), 3.69–3.60 (m, 1H), 2.09 (ddd, *J* = 11.0, 8.6, 4.2 Hz, 2H), 2.02–1.97 (m, 1H), 1.75–1.65 (m, 1H), 1.60–1.56 (m, 2H), 1.56–1.48 (m, 6H), 1.31 (h, *J* = 7.3 Hz, 6H), 1.14–1.08 (m, 6H), 0.86 (t, *J* = 7.3 Hz, 9H). ^13^C NMR (151 MHz, DMSO-*d_6_*) δ 152.0, 150.6, 149.2, 142.7, 136.7, 122.4, 119.4, 113.4, 88.2, 66.9, 30.7, 28.5, 26.6, 24.6, 21.7, 13.5, 10.4. HPLC-MS (ESI): *m*/*z* calcd for C_25_H_41_N_3_OSn 519.23; [M + H]^+^ found 520.35.

#### 3.1.22. 3-(3-Fluorophenyl)-1-(tetrahydro-2*H*-pyran-2-yl)-5-(tributylstannyl)-pyrazole (**15c**)

The synthesis was carried out according to general procedure B (516 mg, 63%). R*_f_*: 0.74 (PE/EtOAc 5:1). ^1^H NMR (600 MHz, DMSO-*d_6_*) δ 7.65 (dt, *J* = 7.7, 1.2 Hz, 1H), 7.59 (ddd, *J* = 10.5, 2.7, 1.5 Hz, 1H), 7.42 (td, *J* = 8.0, 6.2 Hz, 1H), 7.09 (tdd, *J* = 9.1, 2.6, 0.9 Hz, 1H), 6.82 (t, *J* = 4.1 Hz, 1H), 5.30–5.23 (m, 1H), 3.94 (dtd, *J* = 11.2, 3.9, 1.8 Hz, 1H), 3.67–3.58 (m, 1H), 2.10–2.04 (m, 2H), 1.97 (dtd, *J* = 13.2, 4.0, 1.6 Hz, 1H), 1.72–1.64 (m, 1H), 1.59–1.55 (m, 2H), 1.56–1.47 (m, 6H), 1.30 (h, *J* = 7.3 Hz, 6H), 1.13–1.07 (m, 6H), 0.86 (t, *J* = 7.3 Hz, 9H). ^13^C NMR (151 MHz, DMSO-*d_6_*) δ 162.6 (d, *J* = 242.3 Hz), 148.8 (d, *J* = 2.7 Hz), 142.9, 136.0 (d, *J* = 8.3 Hz), 130.6 (d, *J* = 8.5 Hz), 121.3 (d, *J* = 2.7 Hz), 113.9 (d, *J* = 20.9 Hz), 112.6, 111.7 (d, *J* = 22.5 Hz), 88.2, 66.9, 30.7, 28.5, 26.6, 24.6, 21.7, 13.5, 10.3. HPLC-MS (ESI): *m*/*z* calcd for C_21_H_33_FN_2_Sn (THP cleaved by acidic conditions) 452.16; [M+NH_4_]^+^ found 470.40.

#### 3.1.23. 1-(3-Methoxy-7-(3-phenyl-1-(tetrahydro-2*H*-pyran-2-yl)-1*H*-pyrazol-5-yl)-10*H*-phenothiazin-10-yl)ethan-1-one (**16a**)

The synthesis was carried out according to general procedure C (149 mg, 53%). R*_f_*: 0.35 (PE/EtOAc 1:1). ^1^H NMR (600 MHz, DMSO-*d_6_*) δ 7.87 (d, *J* = 7.3 Hz, 2H), 7.75 (d, *J* = 7.6 Hz, 2H), 7.60 (d, *J* = 8.1 Hz, 1H), 7.57 (d, *J* = 8.8 Hz, 1H), 7.43 (t, *J* = 7.7 Hz, 2H), 7.33 (t, *J* = 7.4 Hz, 1H), 7.17 (d, *J* = 2.8 Hz, 1H), 7.02 (s, 1H), 6.97 (dd, *J* = 8.8, 2.8 Hz, 1H), 5.29 (d, *J* = 9.6 Hz, 1H), 4.03 (d, *J* = 7.3 Hz, 1H), 3.79 (s, 3H), 3.59 (t, *J* = 11.3 Hz, 1H), 2.50–2.43 (m, 1H), 2.17 (s, 3H), 1.97 (s, 1H), 1.85 (d, *J* = 13.0 Hz, 1H), 1.66–1.57 (m, 2H), 1.55–1.50 (m, 1H). ^13^C NMR (151 MHz, DMSO-*d_6_*) δ 168.6, 157.7, 149.6, 143.7, 139.3, 133.0, 132.8, 132.5, 131.2, 128.7, 128.1, 128.0, 127.9, 127.7, 127.4, 127.1, 125.3, 113.6, 112.4, 104.4, 83.8, 66.5, 55.7, 29.0, 24.5, 22.7, 22.0. HPLC-MS (ESI): *m*/*z* calcd for C_24_H_19_N_3_O_2_S (THP cleaved by acidic conditions) 413.12; [M + H]^+^ found 414.15.

#### 3.1.24. 1-(3-Methoxy-7-(3-(pyridin-2-yl)-1-(tetrahydro-2*H*-pyran-2-yl)-1*H*-pyrazol-5-yl)-10H-phenothiazin-10-yl)ethan-1-one (**16b**)

The synthesis was carried out according to general procedure C (43.0 mg, 30%). R*_f_*: 0.18 (PE/EtOAc 1:3). ^1^H NMR (600 MHz, DMSO-*d_6_*) δ 8.60 (dt, *J* = 4.8, 1.4 Hz, 1H), 8.00 (dd, *J* = 7.7, 1.2 Hz, 1H), 7.86 (td, *J* = 7.7, 1.9 Hz, 1H), 7.77 (s, 1H), 7.75 (d, *J* = 8.3 Hz, 1H), 7.63–7.60 (m, 1H), 7.57 (d, *J* = 8.9 Hz, 1H), 7.34 (ddd, *J* = 7.5, 4.8, 1.2 Hz, 1H), 7.18 (d, *J* = 2.9 Hz, 1H), 7.08 (s, 1H), 6.97 (dd, *J* = 8.9, 2.9 Hz, 1H), 5.32 (dd, *J* = 11.6, 2.3 Hz, 1H), 4.08–4.01 (m, 1H), 3.79 (s, 3H), 3.65–3.56 (m, 1H), 2.49–2.40 (m, 1H), 2.17 (s, 3H), 2.05–1.95 (m, 1H), 1.87 (d, *J* = 12.8 Hz, 1H), 1.66–1.58 (m, 2H), 1.56–1.51 (m, 1H). ^13^C NMR (151 MHz, DMSO-*d_6_*) δ 168.6, 157.7, 151.4, 150.4, 149.3, 143.8, 139.4, 137.0, 136.9, 131.2, 128.8, 128.7, 128.0, 127.7, 127.5, 127.2, 123.0, 119.4, 113.6, 112.4, 105.3, 83.9, 66.5, 55.7, 29.0, 24.5, 22.7, 22.0. HPLC-MS (ESI): *m*/*z* calcd for C_23_H_18_N_4_O_2_S (THP cleaved by acidic conditions) 414.12; [M + H]^+^ found 415.05.

#### 3.1.25. 1-(3-(3-(3-Fluorophenyl)-1-(tetrahydro-2*H*-pyran-2-yl)-1*H*-pyrazol-5-yl)-7-methoxy-10*H*-phenothiazin-10-yl)ethan-1-one (**16c**)

The synthesis was carried out according to general procedure C (134 mg, 56%). R*_f_*: 0.37 (PE/EtOAc 1:1). ^1^H NMR (600 MHz, DMSO-*d_6_*) δ 7.80–7.73 (m, 2H), 7.72 (dd, *J* = 7.8, 1.2 Hz, 1H), 7.65 (dt, *J* = 10.5, 2.0 Hz, 1H), 7.63–7.58 (m, 1H), 7.57 (d, *J* = 9.0 Hz, 1H), 7.48 (dq, *J* = 8.2, 1.8 Hz, 1H), 7.20–7.14 (m, 2H), 7.11 (s, 1H), 6.98 (dt, *J* = 8.9, 2.6 Hz, 1H), 5.30 (t, *J* = 6.4 Hz, 1H), 4.03 (dt, *J* = 9.9, 3.5 Hz, 1H), 3.79 (s, 3H), 3.60 (s, 1H), 2.46 (t, *J* = 11.3 Hz, 1H), 2.17 (s, 3H), 1.98–1.93 (m, 1H), 1.89–1.81 (m, 1H), 1.62 (h, *J* = 11.6 Hz, 2H), 1.53 (d, *J* = 12.2 Hz, 1H). ^13^C NMR (151 MHz, DMSO-*d_6_*) δ 168.7, 162.6 (d, *J* = 242.6 Hz), 157.7, 148.5 (d, *J* = 2.3 Hz), 144.0, 139.4, 135.3 (d, *J* = 8.4 Hz), 132.6, 131.2, 130.8 (d, *J* = 8.3 Hz), 128.1, 128.0, 128.0, 127.7, 127.4, 127.2, 121.3 (d, *J* = 1.8 Hz), 114.6 (d, *J* = 21.0 Hz), 113.7, 112.4, 111.8 (d, *J* = 22.7 Hz), 104.8, 83.9, 66.6, 55.7, 29.0, 24.5, 22.7, 22.0. HPLC-MS (ESI): *m*/*z* calcd for C_24_H_18_FN_3_O_2_S (THP cleaved by acidic conditions) 431.11; [M + H]^+^ found 432.15.

#### 3.1.26. 1-(3-(2-Fluoroethoxy)-7-(3-phenyl-1*H*-pyrazol-5-yl)-10*H*-phenothiazin-10-yl)ethan-1-one (**17a**)

The synthesis was carried out according to general procedure C (51.0 mg, 20%). R*_f_*: 0.28 (PE/EtOAc 1:2). ^1^H NMR (600 MHz, DMSO-*d_6_*) δ 7.86 (dt, *J* = 7.0, 1.2 Hz, 2H), 7.76 (br s, 2H), 7.61 (d, *J* = 9.0 Hz, 1H), 7.58 (d, *J* = 8.8 Hz, 1H), 7.43 (t, *J* = 7.7 Hz, 2H), 7.34 (tt, *J* = 7.4, 1.2 Hz, 1H), 7.23 (d, *J* = 2.8 Hz, 1H), 7.03 (s, 1H), 7.02 (dd, *J* = 8.8, 2.8 Hz, 1H), 5.29 (d, *J* = 9.6 Hz, 1H), 4.74 (dt, *J* = 47.8, 3.9 Hz, 2H), 4.29 (d, *J* = 31.7 Hz, 2H), 4.03 (dp, *J* = 11.3, 2.1 Hz, 1H), 3.60 (br s, 1H), 2.49–2.43 (m, 1H), 2.17 (s, 3H), 2.01–1.95 (m, 1H), 1.85 (d, *J* = 12.9 Hz, 1H), 1.67–1.57 (m, 2H), 1.56–1.50 (m, 1H). ^13^C NMR (151 MHz, DMSO-*d_6_*) δ 168.7, 156.6, 149.6, 143.7, 139.2, 133.0, 132.9, 132.5, 131.5, 128.7, 128.2, 128.1, 127.9, 127.7, 127.4, 127.2, 125.3, 114.2, 113.1, 104.4, 83.9, 82.0 (d, *J* = 166.6 Hz), 67.6 (d, *J* = 18.8 Hz), 66.5, 29.1, 24.5, 22.7, 22.1. HPLC-MS (ESI): *m*/*z* calcd for C_25_H_20_FN_3_O_2_S (THP cleaved by acidic conditions) 445.13; [M + H]^+^ found 446.10.

#### 3.1.27. 1-(3-(2-Fluoroethoxy)-7-(3-(pyridin-2-yl)-1-(tetrahydro-2*H*-pyran-2-yl)-1*H*-pyrazol-5-yl)-10*H*-phenothiazin-10-yl)ethan-1-one (**17b**)

The synthesis was carried out according to general procedure C (66.0 mg, 29%). R*_f_*: 0.23 (PE/EtOAc 1:5). ^1^H NMR (600 MHz, DMSO-*d_6_*) δ 8.61 (ddd, *J* = 4.9, 1.8, 1.0 Hz, 1H), 8.00 (dt, *J* = 7.9, 1.1 Hz, 1H), 7.86 (td, *J* = 7.7, 1.8 Hz, 1H), 7.78 (s, 1H), 7.75 (d, *J* = 8.1 Hz, 1H), 7.63 (d, *J* = 8.0 Hz, 1H), 7.58 (d, *J* = 8.8 Hz, 1H), 7.35 (ddd, *J* = 7.5, 4.8, 1.2 Hz, 1H), 7.23 (d, *J* = 2.8 Hz, 1H), 7.08 (s, 1H), 7.02 (dd, *J* = 8.8, 2.8 Hz, 1H), 5.32 (d, *J* = 9.6 Hz, 1H), 4.74 (dt, *J* = 47.8, 3.9 Hz, 2H), 4.29 (d, *J* = 29.9 Hz, 2H), 4.04 (d, *J* = 11.4 Hz, 1H), 3.61 (s, 1H), 2.49–2.43 (m, 1H), 2.17 (s, 3H), 1.98 (br s, 1H), 1.85 (br s, 1H), 1.68–1.57 (m, 2H), 1.56–1.52 (m, 1H). ^13^C NMR (151 MHz, DMSO-*d_6_*) δ 168.7, 156.6, 151.4, 150.4, 149.3, 143.8, 139.3, 139.3, 136.9, 131.5, 128.8, 128.7, 128.1, 128.0, 127.7, 127.3, 123.0, 119.5, 114.2, 113.1, 105.3, 83.9, 82.0 (d, *J* = 166.8 Hz), 67.6 (d, *J* = 18.8 Hz), 66.6, 29.0, 24.5, 22.7, 22.0. HPLC-MS (ESI): *m*/*z* calcd for C_24_H_19_FN_4_O_2_S (THP cleaved by acidic conditions) 446.12; [M + H]^+^ found 447.15.

#### 3.1.28. 1-(3-(2-Fluoroethoxy)-7-(3-(3-fluorophenyl)-1*H*-pyrazol-5-yl)-10*H*-phenothiazin-10-yl)ethan-1-one (**17c**)

The synthesis was carried out according to general procedure C (109 mg, 43%). R*_f_*: 0.31 (PE/EtOAc 1:2). ^1^H NMR (600 MHz, DMSO-*d_6_*) δ 13.55 (d, *J* = 10.2 Hz, 1H), 7.99 (d, *J* = 1.8 Hz, 1H), 7.84 (dd, *J* = 39.6, 6.3 Hz, 1H), 7.73–7.66 (m, 2H), 7.66–7.60 (m, 2H), 7.57–7.51 (m, 2H), 7.36 (s, 1H), 7.21 (s, 1H), 7.00 (dd, *J* = 9.3, 2.6 Hz, 1H), 4.74 (dt, *J* = 47.8, 3.9 Hz, 2H), 4.29 (dt, *J* = 29.8, 5.0 Hz, 2H), 2.15 (s, 3H). ^13^C NMR (151 MHz, DMSO-*d_6_*) δ 169.2, 163.7 (d, *J* = 243.3 Hz), 157.0, 150.8 (d, *J* = 13.8 Hz), 142.9, 133.5, 132.8, 132.5, 132.5, 132.0 (d, *J* = 9.9 Hz), 131.6, 129.2 (d, *J* = 11.7 Hz), 128.6, 128.6, 124.5, 124.4, 121.6, 115.3 (d, *J* = 13.6 Hz), 114.5, 113.5, 112.2 (d, *J* = 8.9 Hz), 101.3, 82.5 (d, *J* = 166.7 Hz), 68.1 (d, *J* = 18.9 Hz), 23.1. HPLC-MS (ESI): *m*/*z* calcd for C_25_H_19_F_2_N_3_O_2_S 463.12; [M + H]^+^ found 464.40.

#### 3.1.29. *N*-(2-((3-Methoxyphenyl)thio)-4-(3-phenyl-1-(tetrahydro-2*H*-pyran-2-yl)-1*H*-pyrazol-5-yl)phenyl)acetamide (**18a**)

The synthesis was carried out according to general procedure D (45.0 mg, 29%). R*_f_*: 0.56 (PE/EtOAc 1:1). ^1^H NMR (600 MHz, DMSO-*d_6_*) δ 9.55 (s, 1H), 7.88–7.82 (m, 3H), 7.61–7.55 (m, 2H), 7.42 (t, *J* = 7.7 Hz, 2H), 7.33 (tt, *J* = 7.4, 1.1 Hz, 1H), 7.29 (t, *J* = 8.0 Hz, 1H), 6.93 (s, 1H), 6.88–6.84 (m, 2H), 6.82 (t, *J* = 2.1 Hz, 1H), 5.11 (dd, *J* = 10.0, 2.5 Hz, 1H), 3.85–3.80 (m, 1H), 3.71 (s, 3H), 3.19 (td, *J* = 11.3, 2.6 Hz, 1H), 2.42 (tdd, *J* = 13.5, 9.9, 4.2 Hz, 1H), 2.06 (s, 3H), 1.97–1.92 (m, 1H), 1.82–1.76 (m, 1H), 1.55–1.48 (m, 2H), 1.45–1.40 (m, 1H). ^13^C NMR (151 MHz, DMSO-*d_6_*) δ 168.7, 159.8, 149.6, 143.8, 138.9, 137.4, 136.3, 133.0, 132.8, 130.4, 129.0, 128.5, 127.7, 126.9, 125.3, 125.2, 121.9, 115.3, 112.6, 103.8, 83.8, 66.3, 55.1, 29.0, 24.4, 23.3, 22.0. HPLC-MS (ESI): *m*/*z* calcd for C_24_H_21_N_3_O_2_S (THP cleaved by acidic conditions) 415.14; [M + H]^+^ found 416.25.

#### 3.1.30. *N*-(2-((3-Methoxyphenyl)thio)-4-(3-(pyridin-2-yl)-1-(tetrahydro-2*H*-pyran-2-yl)-1*H*-pyrazol-5-yl)phenyl)acetamide (**18b**)

The synthesis was carried out according to general procedure D (65.0 mg, 21%). R*_f_*: 0.17 (PE/EtOAc 1:1). ^1^H NMR (600 MHz, DMSO-*d_6_*) δ 9.63 (s, 1H), 8.59 (dt, *J* = 4.8, 1.9, 0.9 Hz, 1H), 7.97 (d, *J* = 7.9 Hz, 1H), 7.84 (td, *J* = 7.9, 1.9 Hz, 1H), 7.82 (d, *J* = 8.3 Hz, 1H), 7.58 (dd, *J* = 8.3, 2.2 Hz, 1H), 7.56 (d, *J* = 2.2 Hz, 1H), 7.33 (ddd, *J* = 7.5, 4.8, 1.2 Hz, 1H), 7.29 (t, *J* = 8.0 Hz, 1H), 6.98 (s, 1H), 6.88–6.84 (m, 3H), 5.12 (dd, *J* = 10.0, 2.4 Hz, 1H), 3.82 (dt, *J* = 12.1, 4.2 Hz, 1H), 3.71 (s, 3H), 3.16 (td, *J* = 11.4, 2.5 Hz, 1H), 2.46–2.40 (m, 1H), 2.06 (s, 3H), 1.93 (dd, *J* = 10.1, 3.7 Hz, 1H), 1.80 (dd, *J* = 13.7, 3.1 Hz, 1H), 1.60 (p, *J* = 7.7 Hz, 2H), 1.51 (dd, *J* = 10.1, 2.7 Hz, 1H). ^13^C NMR (151 MHz, DMSO-*d_6_*) δ 168.8, 159.9, 151.4, 150.4, 149.3, 144.0, 138.8, 136.9, 136.3, 131.4, 130.5, 129.0, 128.8, 128.7, 125.6, 123.0, 122.3, 119.4, 115.6, 112.7, 104.8, 84.0, 66.5, 55.2, 29.0, 24.4, 23.3, 22.1. HPLC-MS (ESI): *m*/*z* calcd for C_23_H_20_N_4_O_2_S (THP cleaved by acidic conditions) 416.13; [M + H]^+^ found 417.15.

#### 3.1.31. *N*-(4-(3-(3-Fluorophenyl)-1-(tetrahydro-2*H*-pyran-2-yl)-1*H*-pyrazol-5-yl)-2-((3-methoxyphenyl)thio)phenyl)acetamide (**18c**)

The synthesis was carried out according to general procedure D (29.0 mg, 15%). R*_f_*: 0.55 (PE/EtOAc 1:1). ^1^H NMR (600 MHz, DMSO-*d_6_*) δ 9.55 (s, 1H), 7.87 (d, *J* = 8.3 Hz, 1H), 7.69 (dt, *J* = 7.8, 1.2 Hz, 1H), 7.62 (ddd, *J* = 10.4, 2.7, 1.5 Hz, 1H), 7.59 (d, *J* = 2.0 Hz, 1H), 7.57 (dd, *J* = 8.3, 2.1 Hz, 1H), 7.46 (td, *J* = 8.0, 6.1 Hz, 1H), 7.28 (t, *J* = 8.0 Hz, 1H), 7.15 (tdd, *J* = 9.0, 2.6, 0.9 Hz, 1H), 7.01 (s, 1H), 6.87–6.84 (m, 2H), 6.82 (t, *J* = 2.1 Hz, 1H), 5.12 (dd, *J* = 10.0, 2.4 Hz, 1H), 3.84–3.80 (m, 1H), 3.71 (s, 3H), 3.19 (td, *J* = 11.3, 2.5 Hz, 1H), 2.42 (qd, *J* = 9.9, 6.6 Hz, 1H), 2.06 (s, 3H), 1.96–1.93 (m, 1H), 1.79 (dd, *J* = 13.0, 3.5 Hz, 1H), 1.55–1.49 (m, 2H), 1.44–1.40 (m, 1H). ^13^C NMR (151 MHz, DMSO-*d_6_*) δ 168.7, 162.5 (d, *J* = 242.8 Hz), 159.8, 148.4 (d, *J* = 2.9 Hz), 144.0, 139.0, 136.3, 135.3 (d, *J* = 8.2 Hz), 133.0, 130.6 (d, *J* = 8.4 Hz), 130.4, 129.0, 126.7, 125.3, 124.5, 121.9, 121.2 (d, *J* = 2.3 Hz), 115.3, 114.4 (d, *J* = 21.4 Hz), 112.6, 111.7 (d, *J* = 22.5 Hz), 104.3, 83.9, 66.4, 55.1, 28.9, 24.3, 23.3, 22.0. HPLC-MS (ESI): *m*/*z* calcd for C_24_H_20_FN_3_O_2_S (THP cleaved by acidic conditions) 433.13; [M + H]^+^ found 434.10.

#### 3.1.32. 3-Methoxy-7-(3-phenyl-1H-pyrazol-5-yl)-10H-phenothiazine (DAP1a)

The synthesis was carried out according to general procedure E (43.0 mg, 44%). R*_f_*: 0.55 (PE/EtOAc 1:3). ^1^H NMR (600 MHz, DMSO-*d_6_*) δ 13.16 (d, *J* = 71.1 Hz, 1H), 8.52 (d, *J* = 59.0 Hz, 1H), 7.81 (dd, *J* = 38.5, 7.2 Hz, 2H), 7.51–7.37 (m, 4H), 7.32 (d, *J* = 27.8 Hz, 1H), 7.03 (d, *J* = 13.3 Hz, 1H), 6.72 (d, *J* = 8.2 Hz, 1H), 6.66 (d, *J* = 8.4 Hz, 1H), 6.62 (dd, *J* = 11.3, 2.3 Hz, 1H), 6.61 (d, *J* = 7.6 Hz, 1H), 3.67 (s, 3H). ^13^C NMR (151 MHz, DMSO-*d_6_*) δ 154.7, 151.1, 142.8, 142.3, 134.8, 133.7, 129.0, 128.6, 127.3, 125.0, 124.6, 122.7, 117.0, 116.3, 115.2, 114.2, 113.2, 111.6, 98.5, 55.4. HPLC-MS (ESI): *m*/*z* calcd for C_22_H_17_N_3_OS 371.11; [M + H]^+^ found 372.15.

#### 3.1.33. 3-Methoxy-7-(3-(pyridin-2-yl)-1*H*-pyrazol-5-yl)-10*H*-phenothiazine (**DAP1b**)

The synthesis was carried out according to general procedure E (9.00 mg, 34%). R*_f_*: 0.35 (PE/EtOAc 1:5). ^1^H NMR (600 MHz, DMSO-*d_6_*) δ 13.32 (d, *J* = 117.9 Hz, 1H), 8.60 (s, 1H), 8.52 (d, *J* = 53.3 Hz, 1H), 7.93 (d, *J* = 44.3 Hz, 1H), 7.84 (s, 1H), 7.53–7.28 (m, 3H), 7.16 (d, *J* = 69.4 Hz, 1H), 6.70 (d, *J* = 8.2 Hz, 1H), 6.68–6.45 (m, 3H), 3.67 (s, 3H). ^13^C NMR (151 MHz, DMSO-*d_6_*) δ 154.7, 149.8, 149.4, 149.3, 139.7, 137.2, 134.9, 134.0, 129.4, 124.7, 122.7, 122.6, 119.3, 117.2, 117.1, 115.1, 114.3, 113.2, 111.6, 99.9, 55.4. HPLC-MS (ESI): *m*/*z* calcd for C_21_H_16_N_4_OS 372.10; [M + H]^+^ found 373.05.

#### 3.1.34. 3-(3-(3-Fluorophenyl)-1*H*-pyrazol-5-yl)-7-methoxy-10*H*-phenothiazine (**DAP1c**)

The synthesis was carried out according to general procedure E (26.0 mg, 29%). R*_f_*: 0.34 (PE/EtOAc 1:1). ^1^H NMR (600 MHz, DMSO-*d_6_*) δ 13.24 (d, *J* = 50.7 Hz, 1H), 8.61 (d, *J* = 62.1 Hz, 1H), 7.66 (br s, 1H), 7.62 (d, *J* = 10.4 Hz, 1H), 7.44 (d, *J* = 31.7 Hz, 2H), 7.38 (s, 1H), 7.13 (br s, 1H), 7.11 (s, 1H), 6.72 (d, *J* = 8.2 Hz, 1H), 6.65 (d, *J* = 8.5 Hz, 1H), 6.62 (dd, *J* = 8.8, 2.3 Hz, 1H), 6.60 (d, *J* = 2.7 Hz, 1H), 3.67 (s, 3H). ^13^C NMR (151 MHz, DMSO-*d_6_*) δ 162.6 (d, *J* = 242.7 Hz), 154.7, 150.3, 142.9, 142.4, 134.8, 133.4, 131.0, 130.7 (d, *J* = 8.9 Hz), 124.6, 122.8, 121.1, 117.0, 116.3, 115.2, 114.3, 114.0 (d, *J* = 23.7 Hz), 113.2, 111.6, 111.5, 99.0, 55.4. HPLC-MS (ESI): *m*/*z* calcd for C_22_H_16_FN_3_OS 389.10; [M + H]^+^ found 390.15.

#### 3.1.35. 3-(2-Fluoroethoxy)-7-(3-phenyl-1*H*-pyrazol-5-yl)-10*H*-phenothiazine (**DAP2a**)

The synthesis was carried out according to general procedure E (14.0 mg, 40%). R*_f_*: 0.50 (PE/EtOAc 1:2). ^1^H NMR (600 MHz, DMSO-*d_6_*) δ 13.10 (s, 1H), 8.56 (s, 1H), 7.80 (d, *J* = 7.6 Hz, 2H), 7.49–7.36 (m, 4H), 7.32 (t, *J* = 7.6 Hz, 1H), 7.03 (s, 1H), 6.80–6.56 (m, 4H), 4.68 (dt, *J* = 47.7, 3.9 Hz, 2H), 4.13 (d, *J* = 30.2 Hz, 2H). ^13^C NMR (151 MHz, DMSO-*d_6_*) δ 153.5, 146.9, 143.1, 142.1, 135.4, 132.1, 128.8, 127.7, 127.6, 125.0, 124.6, 122.8, 117.2, 116.2, 115.2, 114.3, 114.1, 112.5, 98.7, 82.2 (d, *J* = 166.8 Hz), 67.6 (d, *J* = 18.8 Hz). HPLC-MS (ESI): *m*/*z* calcd for C_23_H_18_FN_3_OS 403.12; [M + Na]^+^ found 446.10.

#### 3.1.36. 3-(2-Fluoroethoxy)-7-(3-(pyridin-2-yl)-1*H*-pyrazol-5-yl)-10*H*-phenothiazine (**DAP2b**)

The synthesis was carried out according to general procedure E (12.0 mg, 26%). R*_f_*: 0.44 (PE/EtOAc 1:5). ^1^H NMR (600 MHz, DMSO-*d_6_*) δ 13.33 (d, *J* = 118.9 Hz, 1H), 8.61 (d, *J* = 18.9 Hz, 1H), 8.54 (d, *J* = 49.4 Hz, 1H), 7.93 (dt, *J* = 47.1, 7.7 Hz, 1H), 7.83 (t, *J* = 7.7 Hz, 1H), 7.65–7.27 (m, 3H), 7.16 (d, *J* = 76.3 Hz, 1H), 6.68 (d, *J* = 38.2 Hz, 4H), 4.68 (d, *J* = 47.9 Hz, 2H), 4.14 (d, *J* = 30.1 Hz, 2H). ^13^C NMR (151 MHz, DMSO-*d_6_*) δ 153.5 (d, *J* = 24.5 Hz), 152.1 (d, *J* = 28.5 Hz), 150.6, 149.3 (d, *J* = 38.2 Hz), 142.3, 137.0 (d, *J* = 97.0 Hz), 135.6, 135.2, 127.1, 124.7 (d, *J* = 45.7 Hz), 122.9, 122.7 (d, *J* = 12.8 Hz), 119.5 (d, *J* = 114.6 Hz), 117.2 (d, *J* = 34.0 Hz), 116.1 (d, *J* = 62.7 Hz), 115.2, 114.3, 114.1, 112.5, 99.9 (d, *J* = 65.7 Hz), 82.2 (d, *J* = 167.0 Hz), 67.5 (d, *J* = 19.2 Hz). HPLC-MS (ESI): *m*/*z* calcd for C_22_H_17_FN_4_OS 404.11; [M + H]^+^ found 405.15.

#### 3.1.37. 3-(2-Fluoroethoxy)-7-(3-(3-fluorophenyl)-1*H*-pyrazol-5-yl)-10*H*-phenothiazine (**DAP2c**)

The synthesis was carried out according to general procedure E (11.0 mg, 13%). R*_f_*: 0.57 (PE/EtOAc 1:2). ^1^H NMR (600 MHz, DMSO-*d_6_*) δ 13.25 (d, *J* = 59.9 Hz, 1H), 8.56 (d, *J* = 52.0 Hz, 1H), 7.67 (br s, 1H), 7.61 (d, *J* = 10.5 Hz, 1H), 7.56–7.39 (m, 2H), 7.38 (s, 1H), 7.22–7.05 (m, 2H), 6.71 (d, *J* = 8.2 Hz, 1H), 6.65 (s, 3H), 4.68 (dt, *J* = 48.0, 3.8 Hz, 2H), 4.13 (dt, *J* = 30.3, 3.8 Hz, 2H). ^13^C NMR (151 MHz, DMSO-*d_6_*) δ 162.6 (d, *J* = 242.5 Hz), 153.6, 150.1, 143.1, 140.4, 135.2, 134.6, 131.7, 130.7 (d, *J* = 18.8 Hz), 124.7, 122.8, 121.1, 117.3, 117.1, 115.2, 114.3, 114.2 (d, *J* = 7.0 Hz), 114.1, 112.5, 111.5 (d, *J* = 11.9 Hz), 99.0, 82.2 (d, *J* = 166.4 Hz), 67.5 (d, *J* = 19.1 Hz). HPLC-MS (ESI): *m*/*z* calcd for C_23_H_17_F_2_N_3_OS 421.11; [M + MeCN + 2H]^+^ found 464.10.

#### 3.1.38. 2-((3-Methoxyphenyl)thio)-4-(3-phenyl-1*H*-pyrazol-5-yl)aniline (**DAP3a**)

The synthesis was carried out according to general procedure E (23.0 mg, 81%). R*_f_*: 0.42 (PE/EtOAc 1:1). ^1^H NMR (600 MHz, DMSO-*d_6_*) δ 13.02 (s, 1H), 7.84 (d, *J* = 2.1 Hz, 1H), 7.81 (d, *J* = 7.7 Hz, 2H), 7.66 (d, *J* = 8.4 Hz, 1H), 7.42 (t, *J* = 7.6 Hz, 2H), 7.31 (t, *J* = 7.4 Hz, 1H), 7.20 (t, *J* = 8.0 Hz, 1H), 6.99 (s, 1H), 6.90 (d, *J* = 8.4 Hz, 1H), 6.73 (ddd, *J* = 8.0, 2.1, 0.9 Hz, 1H), 6.68 (dt, *J* = 8.0, 2.1, 0.9 Hz, 1H), 6.66 (t, *J* = 2.1 Hz, 1H), 5.57 (s, 2H), 3.69 (s, 3H). ^13^C NMR (151 MHz, DMSO-*d_6_*) δ 159.6, 150.1, 150.0, 143.2, 137.9, 137.8, 133.7, 129.9, 128.6, 128.5, 128.3, 127.4, 125.0, 118.5, 115.0, 112.1, 112.0, 110.8, 98.1, 55.0. HPLC-MS (ESI): *m*/*z* calcd for C_22_H_19_N_3_OS 373.12; [M + H]^+^ found 374.10.

#### 3.1.39. 2-((3-Methoxyphenyl)thio)-4-(3-(pyridin-2-yl)-1*H*-pyrazol-5-yl)aniline (**DAP3b**)

The synthesis was carried out according to general procedure E (21.0 mg, 47%). R*_f_*: 0.42 (PE/EtOAc 1:4). ^1^H NMR (600 MHz, DMSO-*d_6_*) δ 13.27 (d, *J* = 84.4 Hz, 1H), 8.59 (d, *J* = 4.9 Hz, 1H), 7.92 (s, 1H), 7.85 (d, *J* = 2.0 Hz, 2H), 7.69 (d, *J* = 6.1 Hz, 1H), 7.31 (br s, 1H), 7.20 (t, *J* = 8.0 Hz, 1H), 7.10 (d, *J* = 8.1 Hz, 1H), 6.90 (d, *J* = 8.4 Hz, 1H), 6.73 (dd, *J* = 8.4, 2.3 Hz, 1H), 6.70–6.62 (m, 2H), 5.65 (br s, 2H), 3.68 (s, 3H). ^13^C NMR (151 MHz, DMSO-d6) δ 159.7, 152.1, 150.3, 149.2, 148.9, 141.9, 138.0, 136.8, 133.7, 130.0, 128.6, 128.5, 122.6, 119.2, 118.6, 115.1, 112.2, 111.9, 110.8, 99.2, 55.1. HPLC-MS (ESI): *m*/*z* calcd for C_21_H_18_N_4_OS 374.12; [M + H]^+^ found 375.15.

#### 3.1.40. 4-(3-(3-Fluorophenyl)-1*H*-pyrazol-5-yl)-2-((3-methoxyphenyl)thio)aniline (**DAP3c**)

The synthesis was carried out according to general procedure E (16.0 mg, 68%). R*_f_*: 0.45 (PE/EtOAc 1:1). ^1^H NMR (600 MHz, DMSO-*d_6_*) δ 13.10 (s, 1H), 7.84 (d, *J* = 2.0 Hz, 1H), 7.66 (t, *J* = 8.8 Hz, 2H), 7.62 (dt, *J* = 10.6, 2.0 Hz, 1H), 7.46 (q, *J* = 7.0, 6.6 Hz, 1H), 7.20 (t, *J* = 8.2 Hz, 1H), 7.12 (t, *J* = 9.7 Hz, 1H), 7.07 (s, 1H), 6.91 (d, *J* = 8.4 Hz, 1H), 6.73 (dd, *J* = 8.2, 2.4 Hz, 1H), 6.68 (dt, *J* = 8.2, 2.1, 1.0 Hz, 1H), 6.66 (t, *J* = 2.1 Hz, 1H), 5.61 (br s, 2H), 3.69 (s, 3H). ^13^C NMR (151 MHz, DMSO-*d_6_*) δ 162.5 (d, *J* = 242.7 Hz), 159.7, 152.6, 150.2 (d, *J* = 14.8 Hz), 143.7, 137.8, 135.1 (d, *J* = 2.1 Hz), 133.8, 130.6 (d, *J* = 5.3 Hz), 130.1, 129.9, 128.3, 121.0 (d, *J* = 1.8 Hz), 118.5, 115.1, 112.1, 112.0, 111.5 (d, *J* = 23.1 Hz), 111.4, 110.8, 97.2, 55.0. HPLC-MS (ESI): *m*/*z* calcd for C_22_H_18_FN_3_OS 391.12; [M + H]^+^ found 392.10.

### 3.2. Radiochemistry

#### 3.2.1. Tritiation of [^3^H]MODAG−001

[^3^H]MODAG−001 was obtained from RC Tritec AG (Teufen, Switzerland). The tracer was produced via an iridium-catalyzed reaction where up to four tritium atoms were exchanged. A solution of [^3^H]MODAG−001 in EtOH with a molar activity (A_m_) of 2.9 GBq/μmol and a radiochemical purity of >99% was stored at −80 °C until use.

#### 3.2.2. Tritiation of [^3^H]SIL26

A mixture of the *N*-acetylated phenolic precursor (Compound **S1** in Supplementary File S1, 3.00 mg, 10.0 µmol) and Cs_2_CO_3_ (5.00 mg, 15.0 µmol) was dissolved in DMSO (100 µL) and stirred at room temperature for 20 min. A solution of [^3^H]2-fluoroethyl(p-tolyl)benzenesulfonate in EtOAc/Hex 1:4 *v*/*v* (1.69 GBq/µmol; RC Tritec AG, Teufen, Switzerland) was dried at 40 °C and dissolved in DMSO. Then, 200 µL (100 MBq) of the solution was added to the reaction mixture. It was heated at 90 °C for 1 h. The addition of DBU afforded the *N*-deprotected product. The mixture was cooled to room temperature, diluted with water to 1 mL, and purified by preparative HPLC using an Altima C18 3 µm, 4.6 × 100 mm column (Mz-Analysentechnik GmbH, Mainz, Germany; isocratic method: 50% B, 3 mL/min; solvent A: 0.1% TFA in H_2_O; solvent B: MeCN). The desired fractions (retention time: approximately 30 min, total volume: 10 mL, analyzed by liquid scintillation counting—LKB/Wallac 1219 Rackbeta, Mount Waverley, Australia) were diluted with water (10 mL) and passed through a Sep-Pak Light tC18 cartridge (Waters, Milford, MA, USA) preconditioned with EtOH (10 mL), water (20 mL), and air (10 mL). The cartridge containing the trapped product was rinsed with water (10 mL). The product was eluted with 1.1 mL EtOH to afford [^3^H]SIL26 (19 MBq/mL, A_m_: 1.7 GBq/µmol, RCY: 30%, 12 μM, RCP: >98%). Reaction progress monitoring, as well as quality control of the collected fractions, was performed by analytical HPLC using a Luna C18 (2) 100 Å 250 × 4.6 mm column (Phenomenex, Torrance, California, USA) under the following conditions: 70% B, 0–20 min, 1 mL/min; solvent A: 0.1% TFA in H_2_O; solvent B: MeCN. The relevant chromatograms are reported in the Supplementary Information.

### 3.3. Biological Evaluation

#### 3.3.1. Expression, Purification, and Fibrillation of Human αSYN

Human αSYN from a prokaryotic host was expressed in *E. coli*. The DNA construct encoding full-length αSYN was cloned into the pET-22b vector and transformed into BL21 cells, and expression was induced overnight at 20 °C with 0.5 mM IPTG. Following cell harvest by centrifugation, the cells were resuspended in 20 mM Tris-HCl pH 7.6, 25 mM NaCl, and 1× complete protease inhibitor (Roche, Basel, Switzerland). The cells were lysed by sonication, boiled for 15 min, and then cleared by centrifugation at 12,000× *g* for 30 min. The αSYN was captured by anion exchange chromatography (HiTrap Q, GE Healthcare, Chicago, Illinois, USA) and eluted by a 0–1 M NaCl gradient over 20 column volumes. Fractions containing αSYN were pooled, concentrated, and applied to a Superdex 75 SEC column equilibrated in PBS. Fractions containing αSYN were collected and applied to a high-capacity endotoxin removal spin column (Pierce, Waltham, Massachusetts, USA). Following this treatment, the endotoxin level was verified to be below 1.0 EU/mg. Dynamic light scattering was used to verify the monomeric state and monodispersity of the pure αSYN. Fibrillation was based on the protocol from Makky et al. describing the formation of the P91 fibrillar assembly [32]. To prepare P91s, monomeric αSYN was thawed and diluted to 4 mg/mL in 20 mM K_3_PO_4_ pH 9.1. The solution was then transferred to round-bottom tubes sealed with parafilm and placed in an orbital shaker for five days at 37 °C and 1000 rpm. The assemblies were sonicated for 10 min with cycles of 20 s on and 10 s off at 50% amplitude (Q800R3, Qsonica, Newtown, CT, USA), and sonicated P91s were aliquoted in volumes of 10 μL and stored at −80 °C for further use.

#### 3.3.2. Preparation of Aβ_1-42_ Fibrils

The generation of Aβ_1-42_ fibrils was adapted from Bagchi et al. and described by Kuebler et al. [11,18]. Synthetic lyophilized human Aβ_1-42_ peptide (5 mg) with >90% purity (EMC Microcollections, Tuebingen, Germany) was dissolved in DMSO (221.5 µL). A monomeric concentration of 150 µM was obtained by adding deionized water (4.1 mL) and 1 M Tris-HCl (111 µL, pH 7.6). The monomers were incubated in an Eppendorf Thermomixer at 37 °C with shaking at 800 rpm for 72 h to induce aggregation. Generated fibrils were sonicated for three minutes in a water bath (Elmasonic S 60 H, Elma Schmidbauer GmbH, Singen, Germany). The final products were aliquoted, frozen on dry ice, and stored at −80 °C until use.

#### 3.3.3. Fibril Binding Assays

To determine the *K*_d_ values of [^3^H]SIL26 and [^3^H]MODAG−001, saturation binding assays were performed on human recombinant αSYN (35 nM for [^3^H]SIL26, 50 nM for [^3^H]MODAG−001) and synthetic human Aβ_1-42_ fibrils (1 µM for both tracers) diluted in phosphate-buffered saline (PBS; Gibco DPBS, no calcium, no magnesium, Thermo Fisher Scientific, Waltham, MA, USA). The fibrils were incubated in 96-well micro test low-binding plates (Ratiolab GmbH, Dreieich, Germany) with increasing concentrations of [^3^H]SIL26 (up to 64 nM) and [^3^H]MODAG−001 (up to 36 nM) in 30 mM Tris-HCl, 0.1% bovine serum albumin, and 0.05% Tween20 in a total volume of 200 µL/well. The tracer was co-incubated with 500 nM non-radioactive SIL26 or MODAG−001 dissolved in DMSO (DMSO concentration ≤0.05% in the final assay) to determine non-specific binding.

Competition binding assays of [^3^H]SIL26 and [^3^H]MODAG−001 were used to determine the binding affinity (*K*_i_ values) of DAP compounds. The test compounds were dissolved in DMSO to a stock concentration of 1 mM, which yielded ≤0.25% DMSO concentration in the final assay. Increasing concentrations of the test compounds (0.06–2500 nM) competed against [^3^H]SIL26 (7 nM for αSYN, 7 nM or 14 nM for Aβ_1-42_) and 1 nM [^3^H]MODAG−001 (for both αSYN and Aβ_1-42_). The concentrations of αSYN fibrils and Aβ_1-42_ fibrils as above were used.

Plates were incubated on a shaker (MaxQ 6000, Thermo Fisher Scientific Inc., Marietta, OH, USA) at 50 rpm for 2 h at 37 °C, covered by removable sealing tape (PerkinElmer, Waltham, MA, USA). Vacuum filtration and read-out were performed as previously reported [18]. Briefly, a glass fiber Filtermat B (PerkinElmer, Waltham, MA, USA) incubated with 0.5% polyethylenimine (PEI; Sigma Aldrich Chemie GmbH, Munich, Germany) for 30 min at 4 °C prior to filter harvesting was used with a FilterMate Harvester (PerkinElmer, Waltham, MA, USA). Melt-on scintillator sheets (MeltiLex B/HS, PerkinElmer, Waltham, MA, USA) were melted onto dried filters. Accumulation of tritium was counted in a liquid scintillation Wallac MicroBeta TriLux counter (PerkinElmer, Waltham, MA, USA) at two minutes/well.

Radioactivity was plotted against increasing concentrations of [^3^H]MODAG−001, [^3^H]SIL26, or the unlabeled test compounds. Data points were fitted using non-linear regression analysis in GraphPad Prism (GraphPad Software, Inc., Version 8.4.0, San Diego, CA, USA).

## 4. Conclusions

Urged by the unmet clinical need for an αSYN PET tracer, this study employed a molecular hybridization strategy to merge two promising scaffolds with the aim of enhancing the compounds’ overall binding properties.

This approach did not afford an improved αSYN ligand but instead significantly reduced the affinity compared to the parent compounds, shifting selectivity towards Aβ binding. Since the αSYN/Aβ selectivity is a critical factor in developing a PET tracer for the aforementioned target, any additional understanding of the structural features responsible for this shift will be pivotal in advancing the field, along with providing new insights on Aβ binding.

Due to their favorable affinity to Aβ, further studies will be needed to investigate the ability of DAP hybrid compounds to inhibit Aβ aggregation and disaggregate preformed fibrils to evaluate their putative theranostic potential.

## Figures and Tables

**Table 1:** The binding affinities of DAP hybrid compounds determined by competition assays against [^3^H]SIL26 and [^3^H]MODAG−001 (mean *K*_i_ ± SEM, three data points).

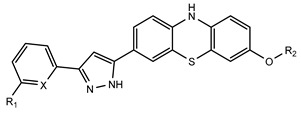							
#	R_1_	R_2_	X	αSYN—*K*_i_ (nM)		Aβ—*K*_i_ (nM)	
				[^3^H]SIL26	[^3^H]MODAG−001	[^3^H]SIL26	[^3^H]MODAG−001
**DAP1a**	H	CH_3_	CH	186.3 ± 144.9	289.3 ± 158.8	10.7 ^b^	10.5 ± 0.9
**DAP1b**	H	CH_3_	N	90.1 ± 56.8	69.9 ± 14.9	7.6 ^b^	10.3 ± 0.8
**DAP1c**	F	CH_3_	CH	>400	>400	1.0 ^b^	15.9 ± 2.4
**DAP2a**	H	CH_2_CH_2_F	CH	>400	143.8 ± 49.5	25.8 ± 5.8 ^a^	7.7 ± 1.7
**DAP2b**	H	CH_2_CH_2_F	N	>400	106.9 ± 31.4	65.8 ± 56.4 ^a^	12.2 ± 1.7
**DAP2c**	F	CH_2_CH_2_F	CH	>400	212.2 ± 93.8	3.1 ± 2.1 ^a^	8.0 ± 1.3

^a^ Two data points available. ^b^ Single data point available.

**Table 2:** The binding affinities of ring-open DAP compounds determined by competition assays against [^3^H]SIL26 and [^3^H]MODAG−001 (mean *K*_i_ ± SEM, three data points).

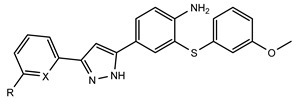						
#	R	X	αSYN—*K*_i_ (nM)		Aβ—*K*_i_ (nM)	
			[^3^H]SIL26	[^3^H]MODAG−001	[^3^H]SIL26	[^3^H]MODAG−001
**DAP3a**	H	CH	no competition ^a^	no competition	86.1 ^b^	>400
**DAP3b**	H	N	no competition ^a^	no competition	n.a.	>400
**DAP3c**	F	CH	no competition ^a^	no competition	n.a.	>400

^a^ Two data points available. ^b^ Single data point available.

## Data Availability

The data presented in this study are available on request from the corresponding author.

## Supplementary Materials

The following supporting information can be downloaded at https://www.mdpi.com/article/10.3390/molecules28104001/s1, Supplementary File S1: HPLC-MS chromatograms of compounds DAP1a-3c, tritium labeling of [^3^H]SIL26, and ^1^H NMR spectra of compounds DAP1a-3c.

## Sample Availability

Samples of the compounds are not available from the authors.
