# Supplementary material for: The Structural Combination of SIL and MODAG Scaffolds Fails to Enhance Binding to α-Synuclein but Reveals Promising Affinity to Amyloid β
Source: Molecules. 2023 May 10;28(10):4001. doi: 10.3390/molecules28104001 (PMC10224536; doi:10.3390/molecules28104001)
Supplement: Supplementary file 1 [file molecules-28-04001-s001.zip › molecules-2343267-Supplementary File S1.pdf]

Supplementary information

Structural combination of SIL and MODAG scaffolds fails to enhance binding to α-synuclein but reveals promising affinity to amyloid β

Di Nanni, A.[1]; Saw, R. S.[1]; Bowden, G. D.[1,2]; Bidesi, N. S. R.[3]; Bjerregaard-Andersen, K.[4]; Korat, Š.[5,6]; Herth, M. M.[3,7]; Pichler, B. J.[1,2]; Herfert, K.[1]; Maurer, A.[1,2,*]

1 Werner Siemens Imaging Center, Department of Preclinical Imaging and Radiopharmacy, Eberhard Karls University Tübingen, Röntgenweg 11-17, 72076 Tübingen, Germany

2 Cluster of Excellence iFIT (EXC 2180) "Image-Guided and Functionally Instructed Tumor Therapies", Eberhard Karls University Tübingen, 72076 Tübingen, Germany

3 Department of Drug Design and Pharmacology, Faculty of Health and Medicinal Sciences, University of Copenhagen, Jagtvej 160, 2100 Copenhagen, Denmark

4 Department of Antibody Engineering and Biochemistry, H. Lundbeck A/S, Ottiliavej 9, 2500 Valby, Denmark

5 Department of Radiology and Nuclear Medicine, Amsterdam UMC, Vrije Universiteit Amsterdam, De Boelelaan 1117, Amsterdam, The Netherlands

6 Amsterdam Neuroscience, Brain Imaging, Amsterdam, The Netherlands

7 Department of Clinical Physiology, Nuclear Medicine & PET, Rigshospitalet, Blegdamsvej 9, 2100 Copenhagen, Denmark

Table of Contents

HPLC-MS chromatograms of compounds DAP1a-3c 2

Tritium labeling of SIL26 7

^{1}H NMR spectra of compounds DAP1a-DAP3c 9

HPLC-MS chromatograms of compounds DAP1a-3c

Chromatograms of compounds DAP1a-DAP3c at 254 nm and mass spectrum correspondent to the main peak (M+1 for DAP1a-c, DAP2b, DAP3a-c; M+23 [Na^+] for DAP2a; M+43 [$MeCN + 2H^+$] for DAP2c).

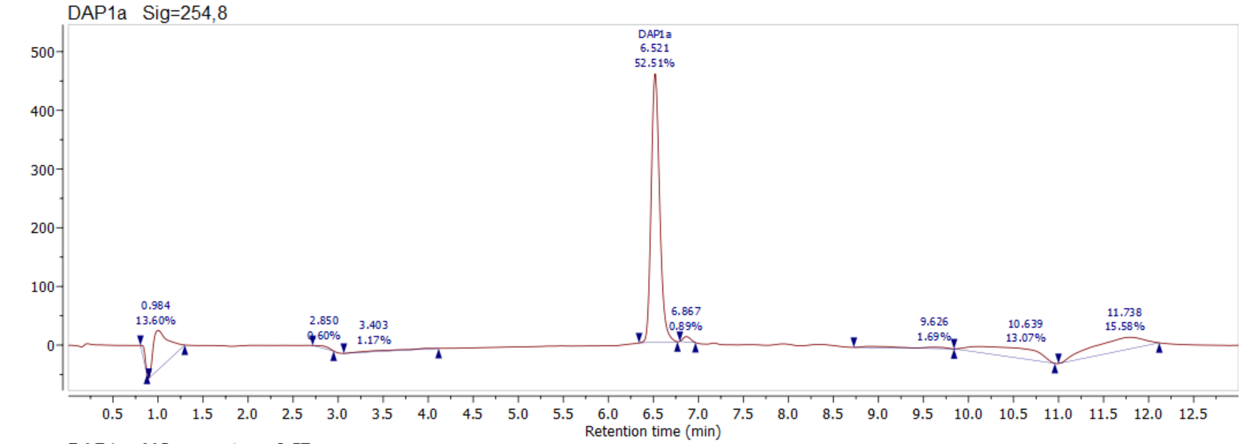

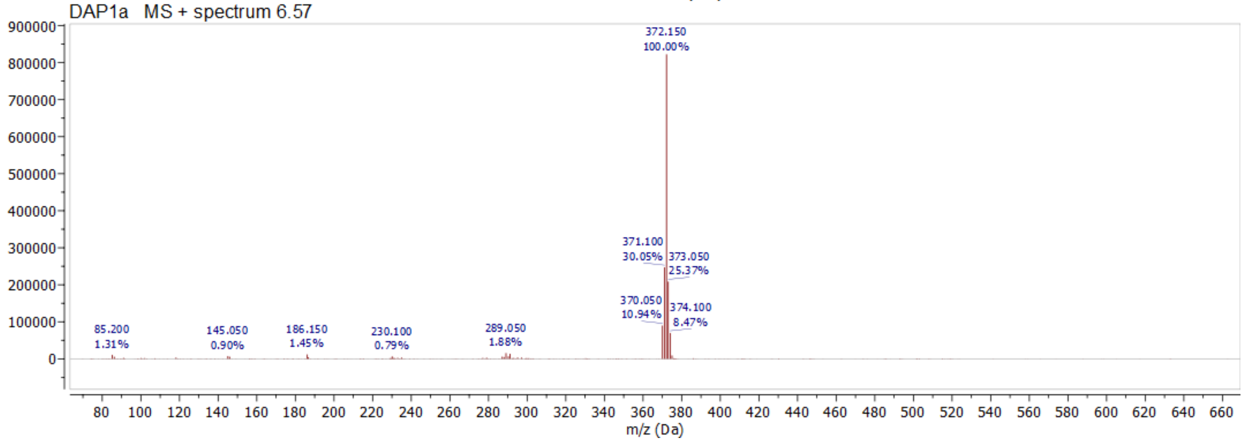

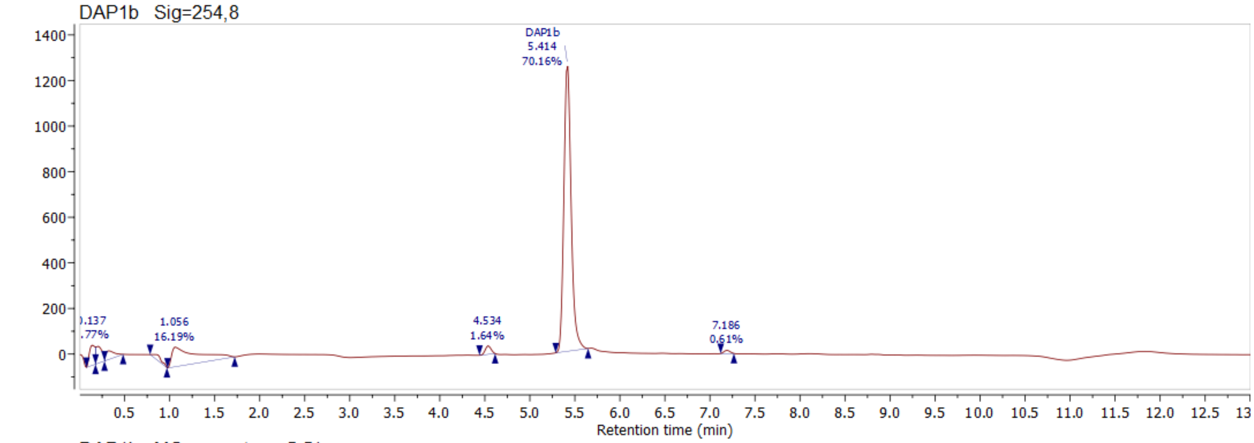
DAP1b Sig=254,8
DAP1b
5.414
70.16%
1.056
16.19%
4.534
1.64%
7.186
0.61%
Retention time (min)

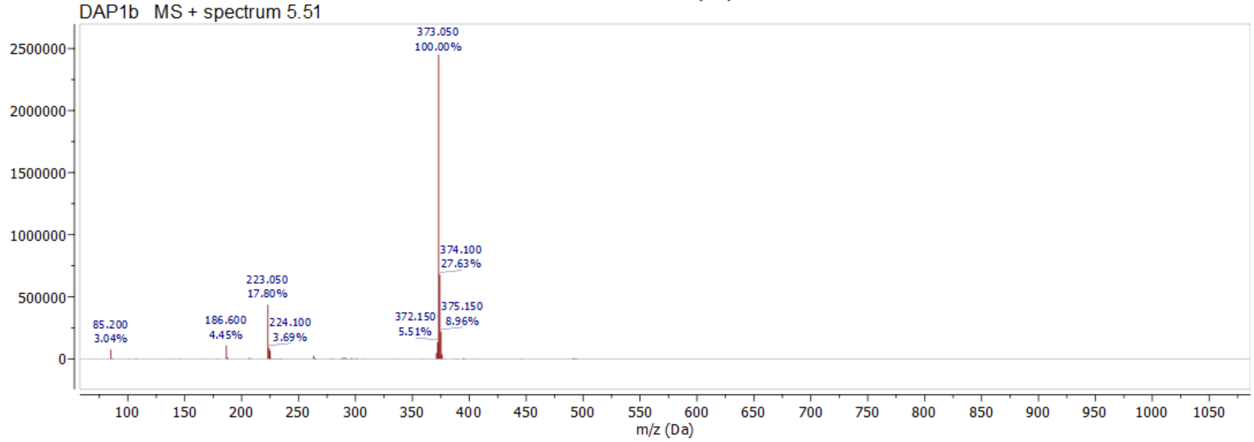
DAP1b MS + spectrum 5.51
373.050
100.00%
374.100
27.63%
223.050
17.80%
375.150
8.96%
372.150
5.51%
85.200
3.04%
186.600
4.45%
224.100
3.69%
m/z (Da)

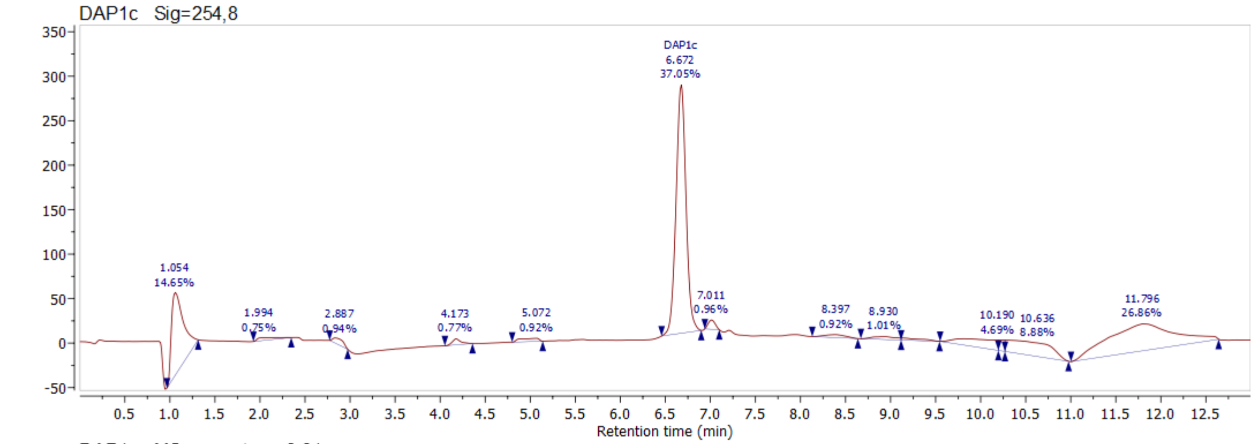
DAP1c Sig=254,8
DAP1c
6.672
37.05%
1.054
14.65%
1.994
0.75%
2.887
0.94%
4.173
0.77%
5.072
0.92%
7.011
0.96%
8.397
0.92%
8.930
1.01%
10.190
4.69%
10.636
8.88%
11.796
26.86%
Retention time (min)

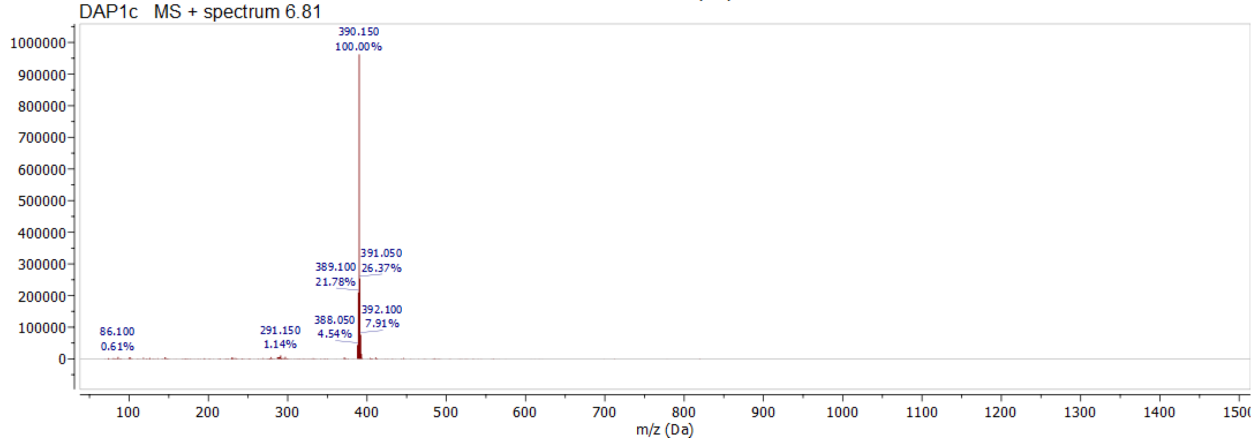
DAP1c MS + spectrum 6.81
390.150
100.00%
391.050
26.37%
389.100
21.78%
392.100
7.91%
388.050
4.54%
291.150
1.14%
86.100
0.61%
m/z (Da)

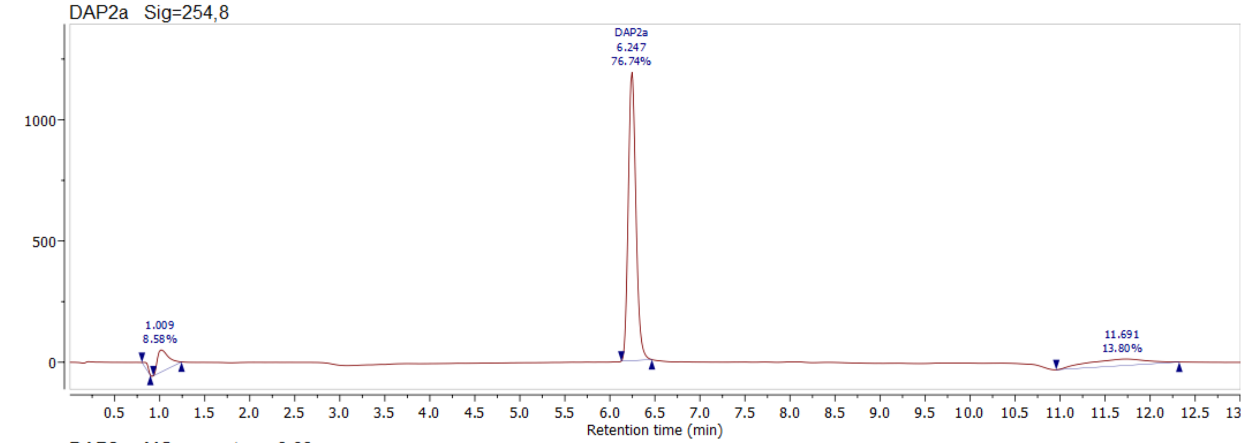
DAP2a Sig=254,8
DAP2a
6.247
76.74%
1.009
8.58%
11.691
13.80%
Retention time (min)

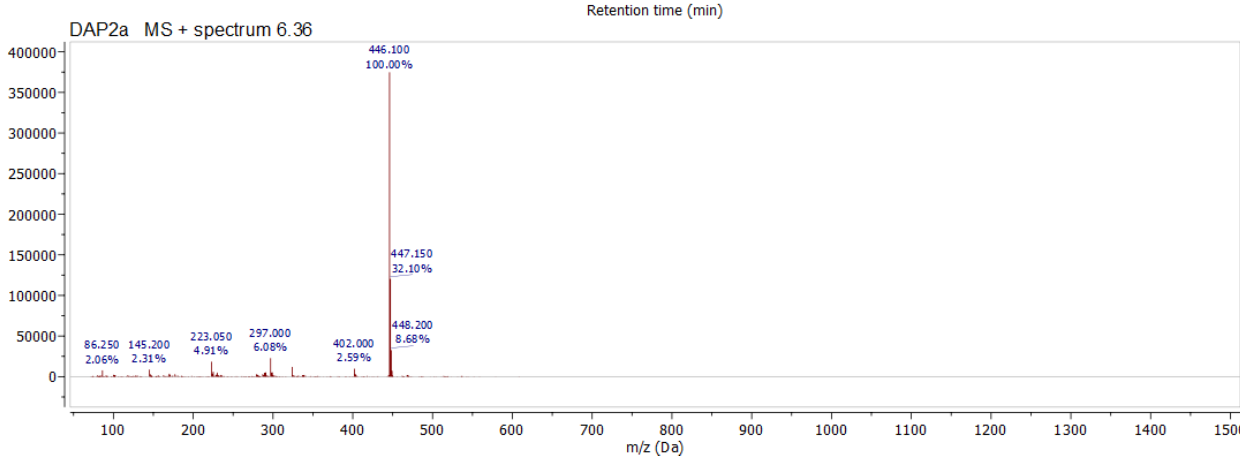
DAP2a MS + spectrum 6.36
446.100
100.00%
447.150
32.10%
448.200
8.68%
86.250
2.06%
145.200
2.31%
223.050
4.91%
297.000
6.08%
402.000
2.59%
m/z (Da)

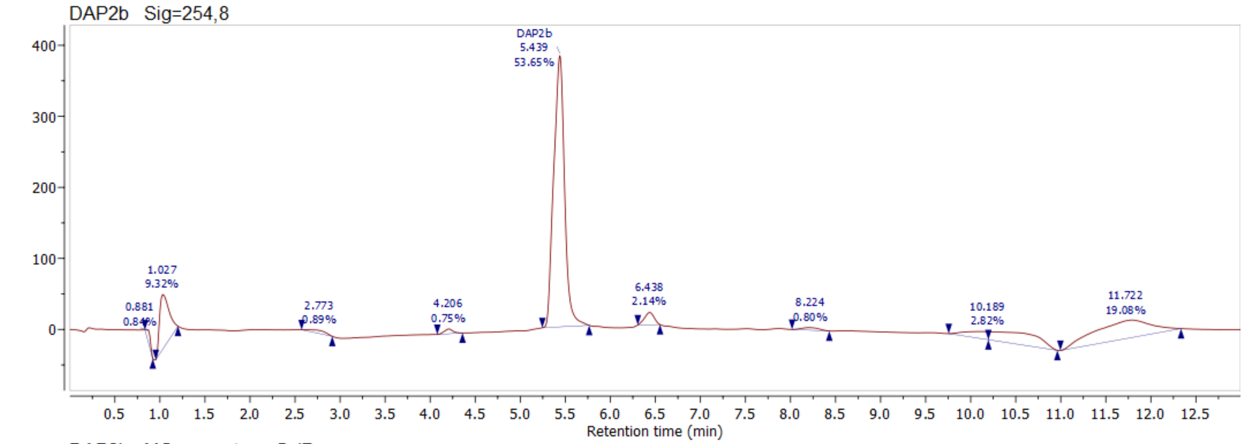
DAP2b Sig=254,8
DAP2b
5.439
53.65%
1.027
9.32%
0.881
0.84%
2.773
0.89%
4.206
0.75%
6.438
2.14%
8.224
0.80%
10.189
2.82%
11.722
19.08%
Retention time (min)

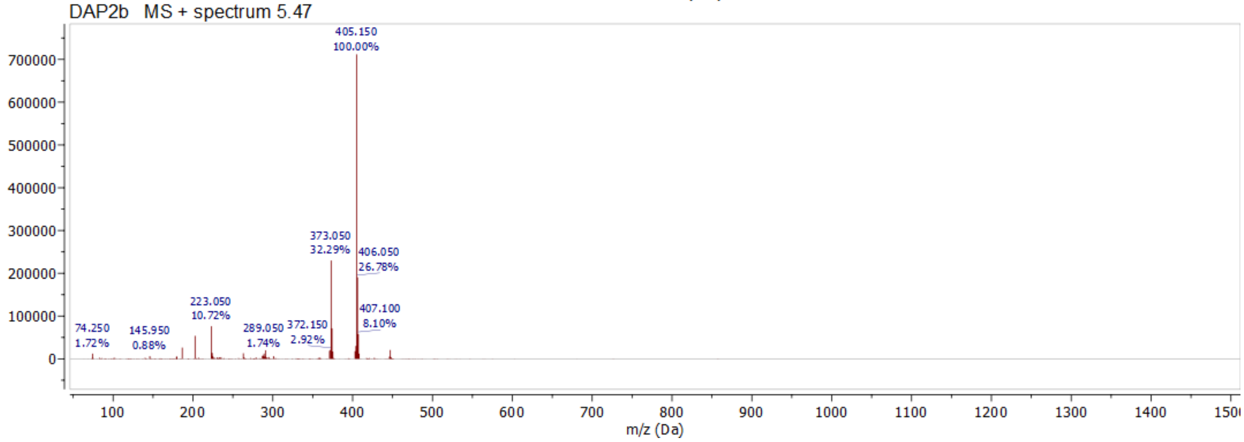
DAP2b MS + spectrum 5.47
405.150
100.00%
373.050
32.29%
406.050
26.78%
407.100
8.10%
223.050
10.72%
74.250
1.72%
145.950
0.88%
289.050
1.74%
372.150
2.92%
m/z (Da)

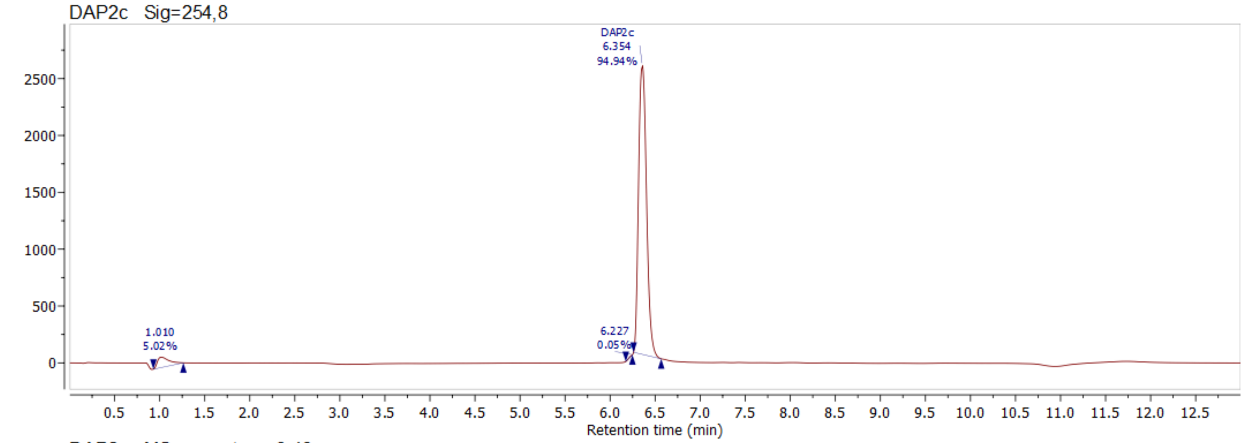
DAP2c Sig=254,8
DAP2c
6.354
94.94%
1.010
5.02%
6.227
0.05%
Retention time (min)

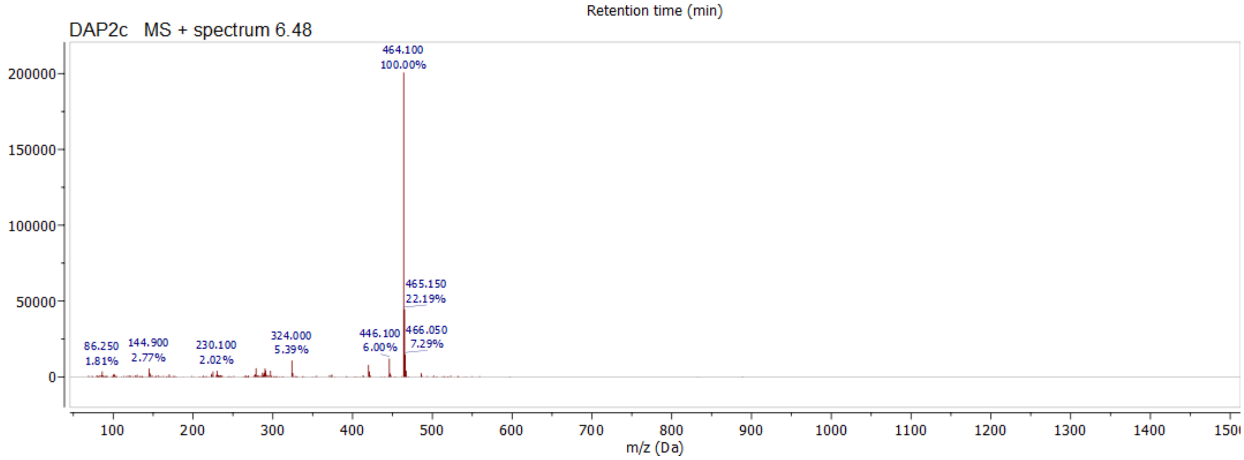
DAP2c MS + spectrum 6.48
464.100
100.00%
465.150
22.19%
466.050
7.29%
446.100
6.00%
324.000
5.39%
230.100
2.02%
144.900
2.77%
86.250
1.81%
m/z (Da)

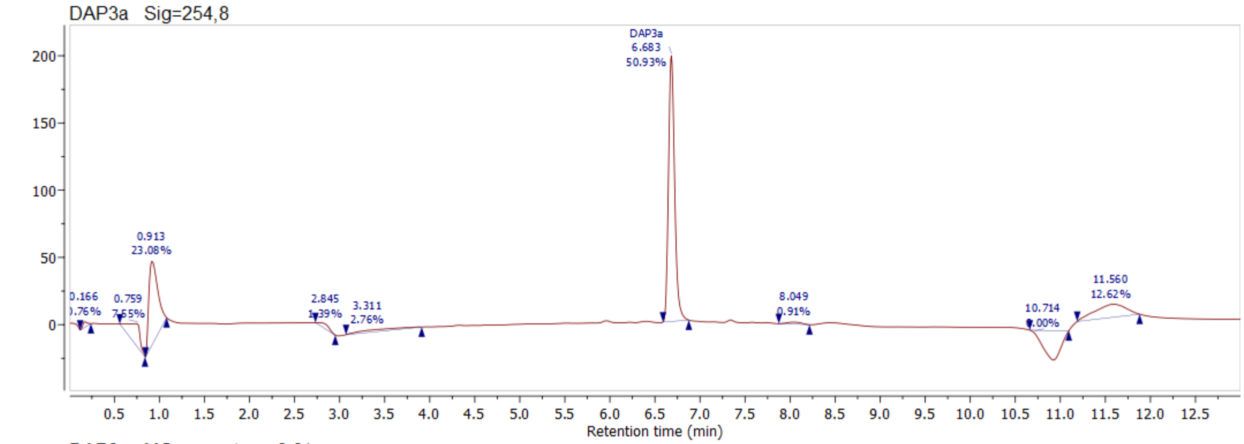
DAP3a Sig=254,8
DAP3a
6.683
50.93%
0.913
23.08%
0.759
2.845
3.311
2.76%
8.049
0.91%
10.714
11.560
12.62%
Retention time (min)

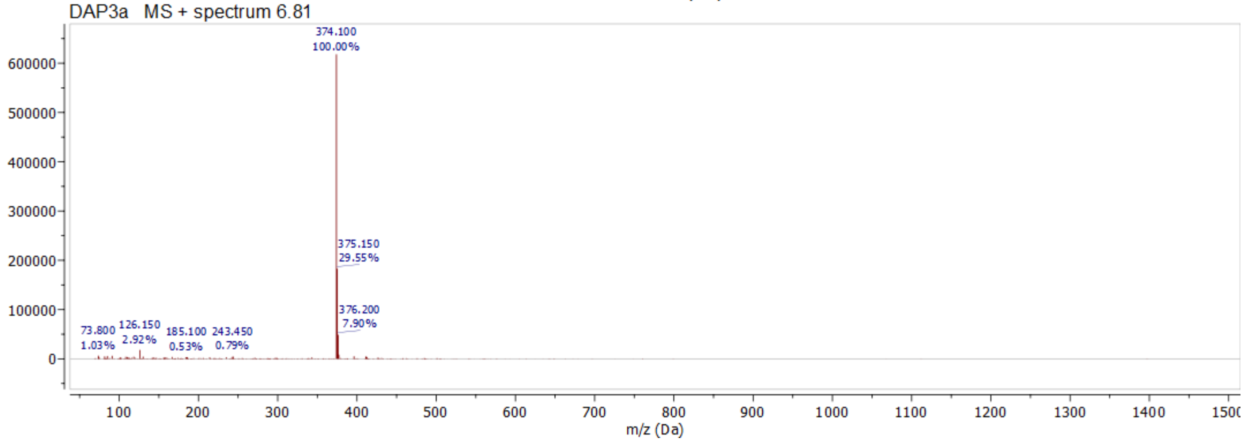
DAP3a MS + spectrum 6.81
374.100
100.00%
375.150
29.55%
376.200
7.90%
73.800
1.03%
126.150
2.92%
185.100
0.53%
243.450
0.79%
m/z (Da)

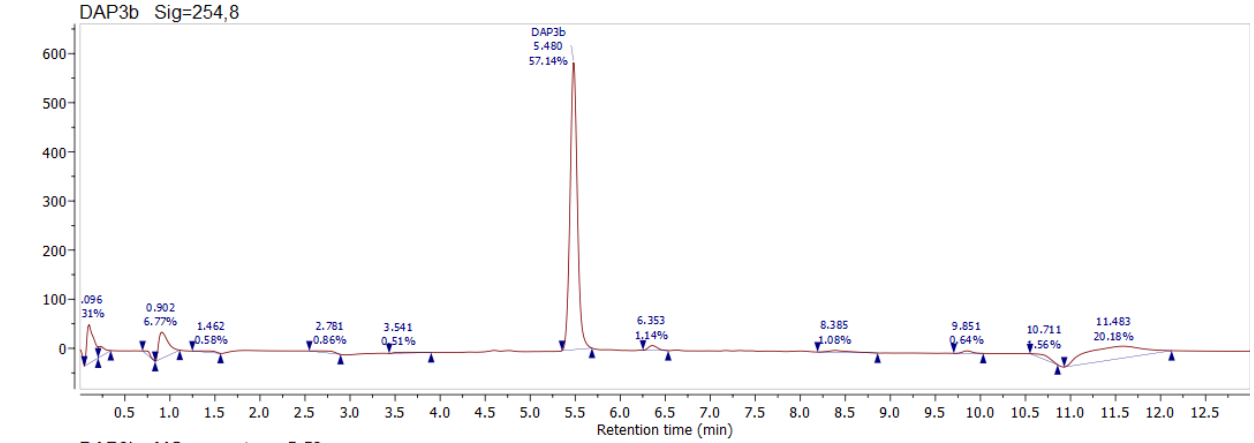
DAP3b Sig=254,8
DAP3b
5.480
57.14%
.096
31%
0.902
6.77%
1.462
0.58%
2.781
0.86%
3.541
0.51%
6.353
1.14%
8.385
1.08%
9.851
0.64%
10.711
.56%
11.483
20.18%
Retention time (min)

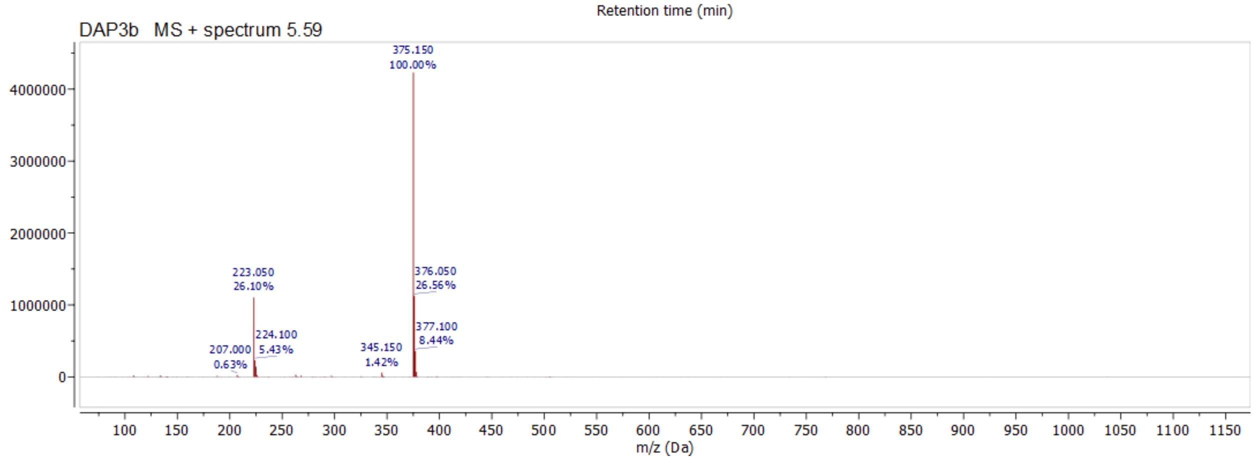
DAP3b MS + spectrum 5.59
375.150
100.00%
223.050
26.10%
376.050
26.56%
377.100
8.44%
224.100
5.43%
207.000
0.63%
345.150
1.42%
m/z (Da)

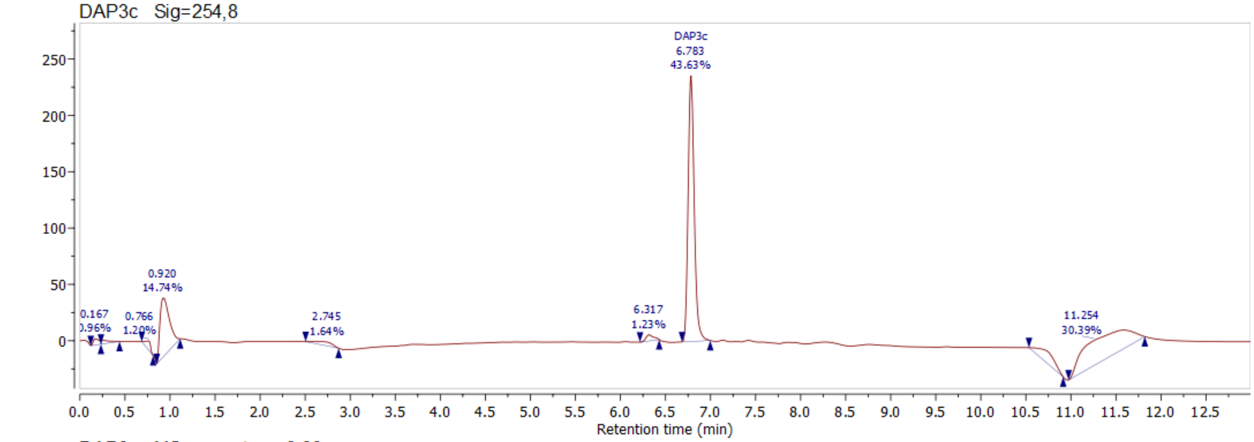
DAP3c Sig=254,8
DAP3c
6.783
43.63%
0.920
14.74%
0.167
0.96%
0.766
1.20%
2.745
1.64%
6.317
1.23%
11.254
30.39%
Retention time (min)

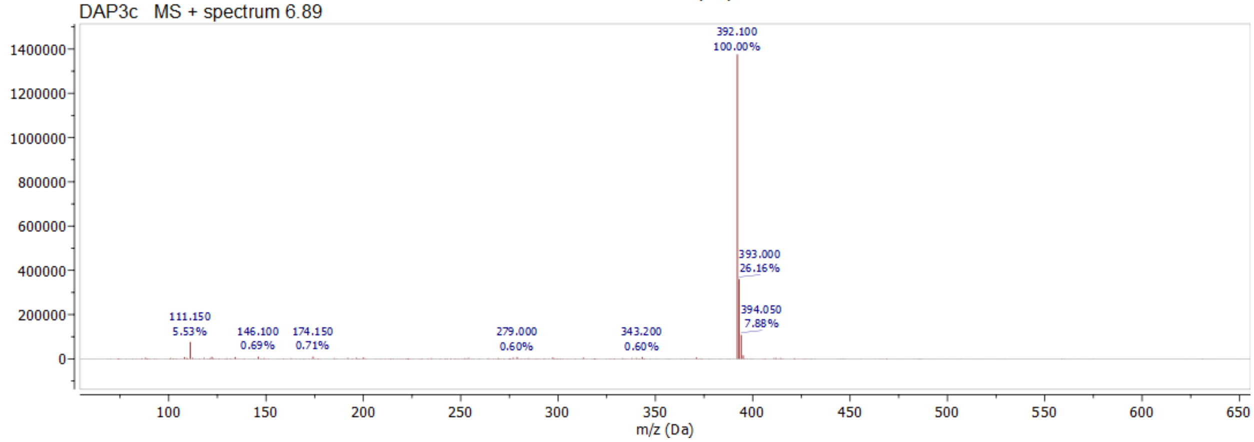
DAP3c MS + spectrum 6.89
392.100
100.00%
393.000
26.16%
394.050
7.88%
111.150
5.53%
146.100
0.69%
174.150
0.71%
279.000
0.60%
343.200
0.60%
m/z (Da)

Tritium labeling of SIL26

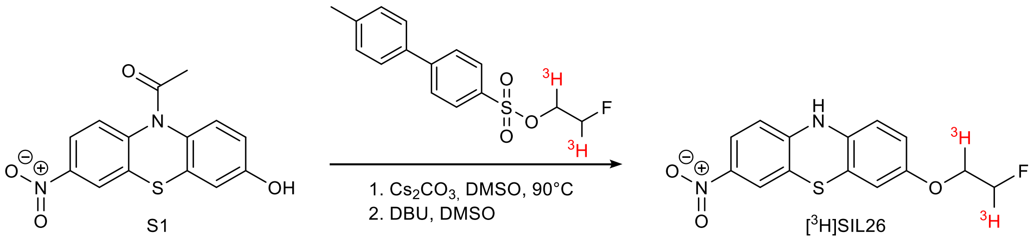

Radiolabeling of [^{3}H]SIL26 from its N-acetylated phenolic precursor.

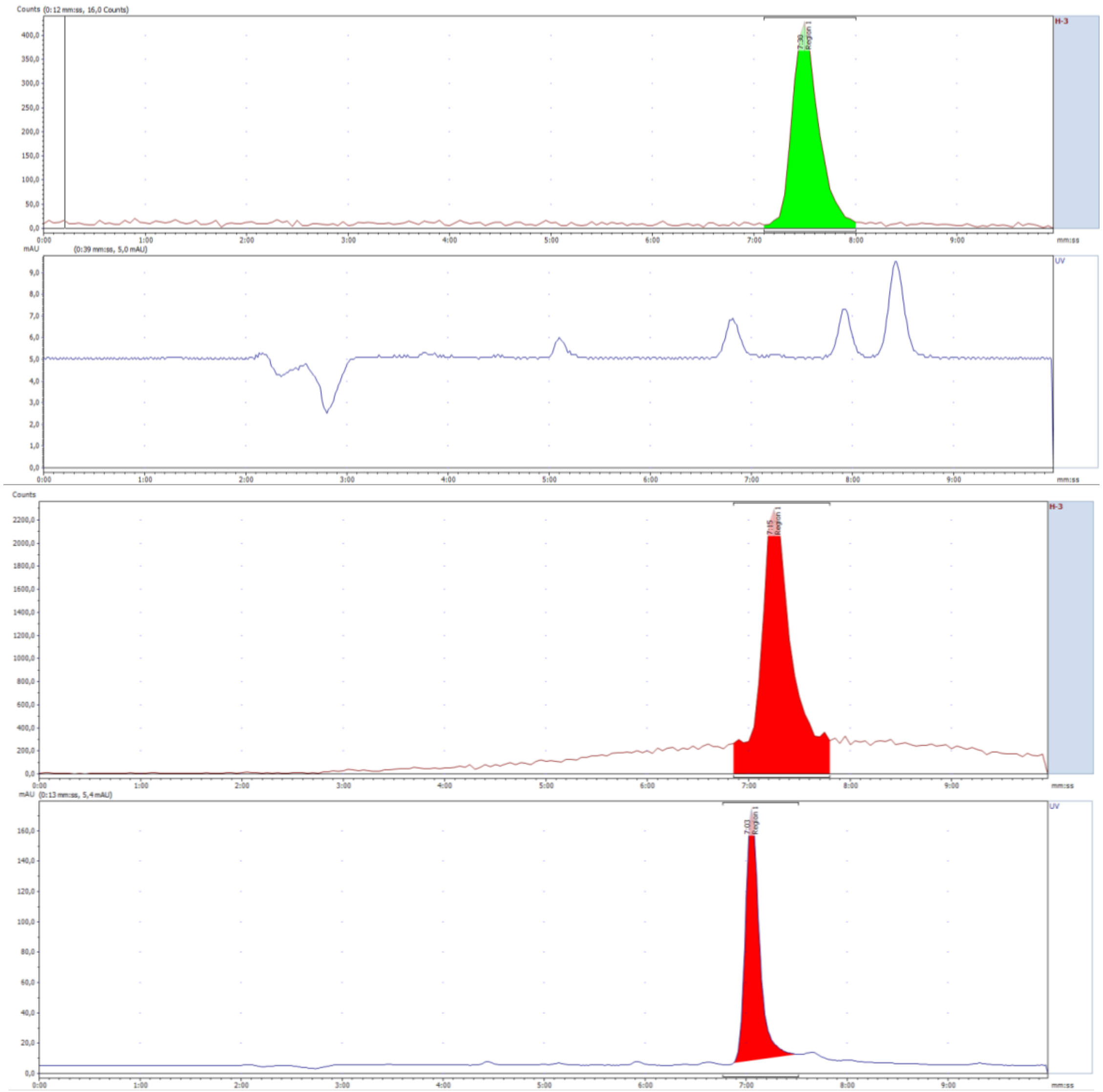

Radio-HPLC chromatogram of formulated [^{3}H]SIL26 (top) and identification of the product by co-injection with non-radioactive SIL26 (bottom). HPLC analysis was performed using an Agilent 1200 Series auto injector (Agilent Technologies, Waldbronn, Germany) and a single Jasco PU-2080Plus pump (Jasco Ltd., Great Dunmow, UK) with an analytical C18 column (Luna C18(2), 100 Å, 250 x 4.6 mm), flowrate: 1 mL/min for elution; 3 mL/min for the scintillation liquid. Detection was performed via a Jasco UV-2075Plus absorbance UV/vis spectrophotometer set at 254 nm and a β-Ram radiochemical detector (LabLogic, Sheffield, UK), using a splitter. HPLC Samples were prepared by diluting 3 µL of the reaction mixture with 500 µL of mobile phase (1:1 MeCN:H_2O), injection volume: 100 µL.

1

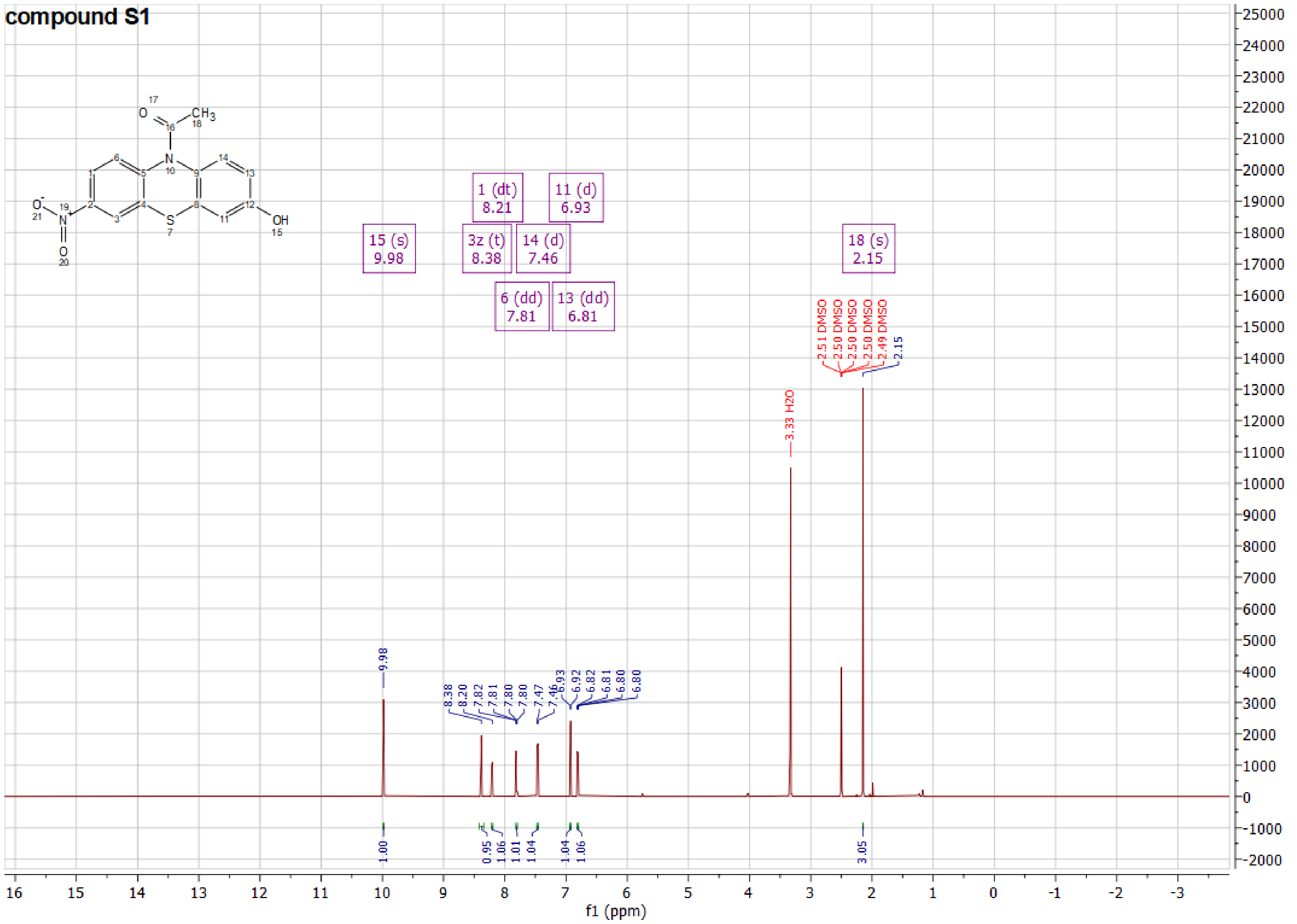
compound S1
15 (s) 9.98
1 (dt) 8.21
3z (t) 8.38
14 (d) 7.46
6 (dd) 7.81
11 (d) 6.93
13 (dd) 6.81
18 (s) 2.15
3.33 H2O
2.51 DMSO
2.50 DMSO
2.50 DMSO
2.50 DMSO
2.49 DMSO
f1 (ppm)

^{1}H NMR spectra of compounds DAP1a-DAP3c

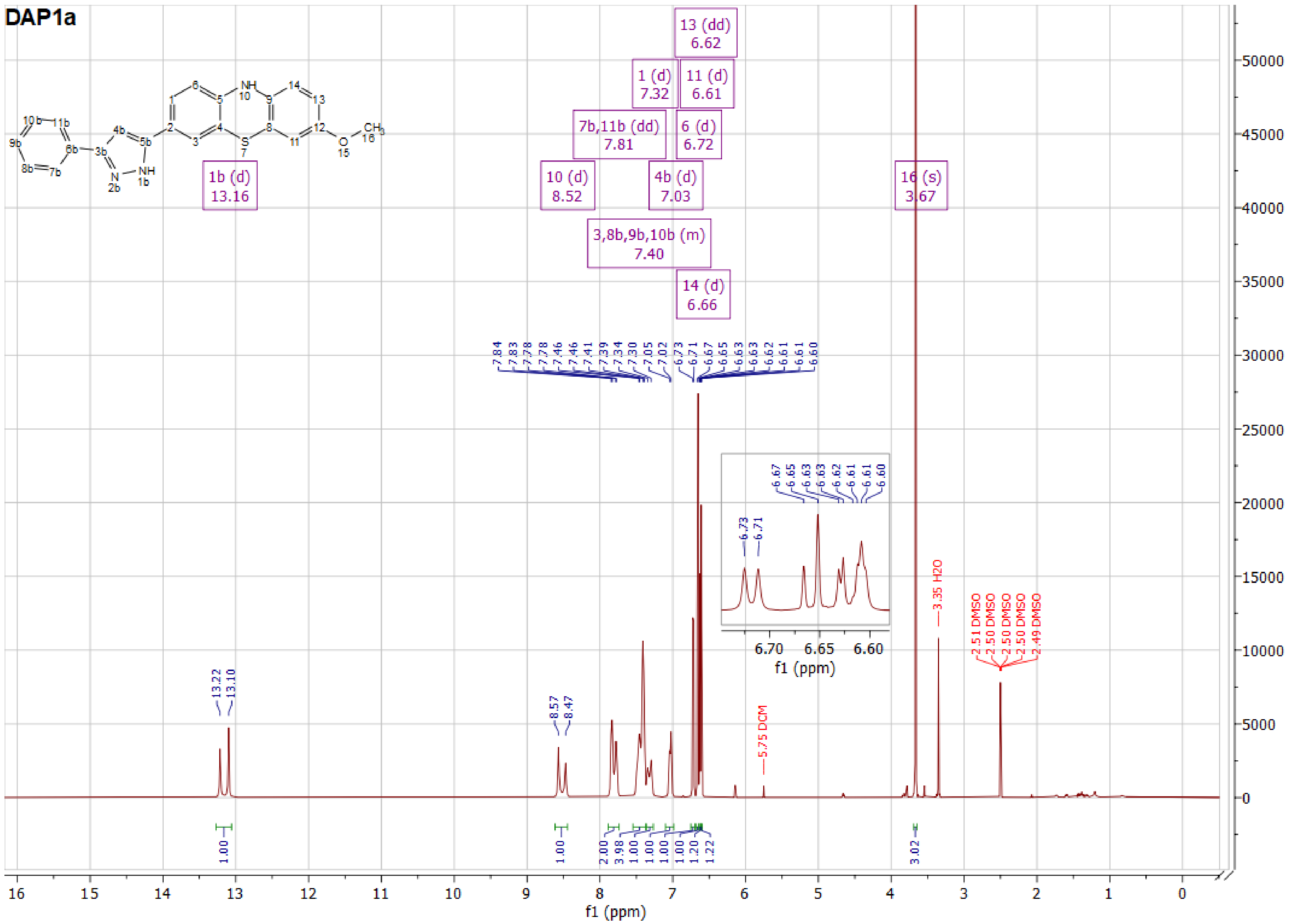

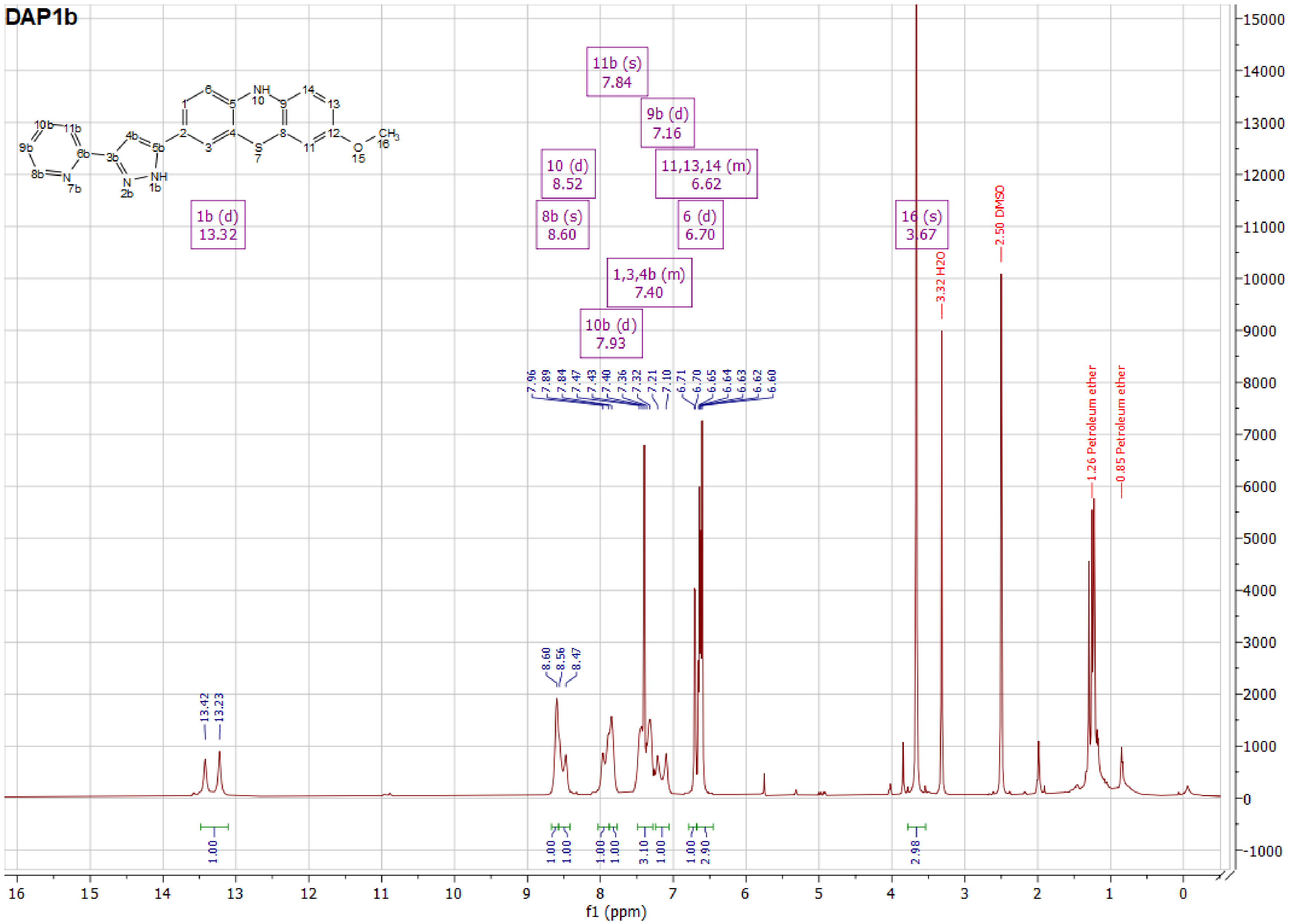

DAP1b
1b (d)
13.32
8b (s)
8.60
10 (d)
8.52
11b (s)
7.84
9b (d)
7.16
11,13,14 (m)
6.62
6 (d)
6.70
1,3,4b (m)
7.40
10b (d)
7.93
16 (s)
3.67
3.32 H2O
2.50 DMSO
1.26 Petroleum ether
0.85 Petroleum ether
f1 (ppm)

4

5

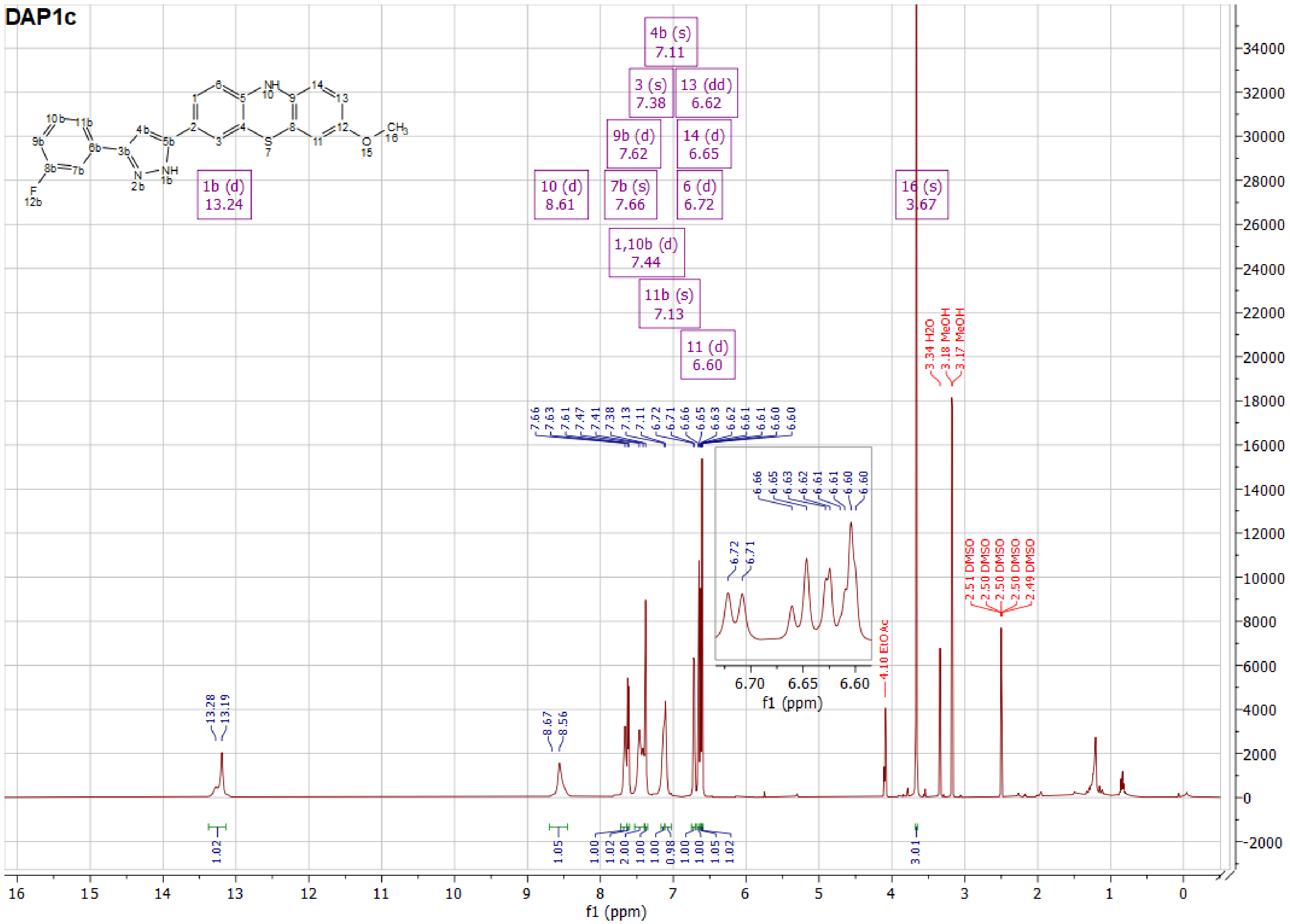

DAP1c
1b (d) 13.24
10 (d) 8.61
7b (s) 7.66
9b (d) 7.62
1,10b (d) 7.44
3 (s) 7.38
11b (s) 7.13
4b (s) 7.11
6 (d) 6.72
14 (d) 6.65
13 (dd) 6.62
11 (d) 6.60
16 (s) 3.67
4.10 EtOAc
3.34 H2O
3.18 MeOH
3.17 MeOH
2.51 DMSO
2.50 DMSO
2.50 DMSO
2.50 DMSO
2.49 DMSO
f1 (ppm)

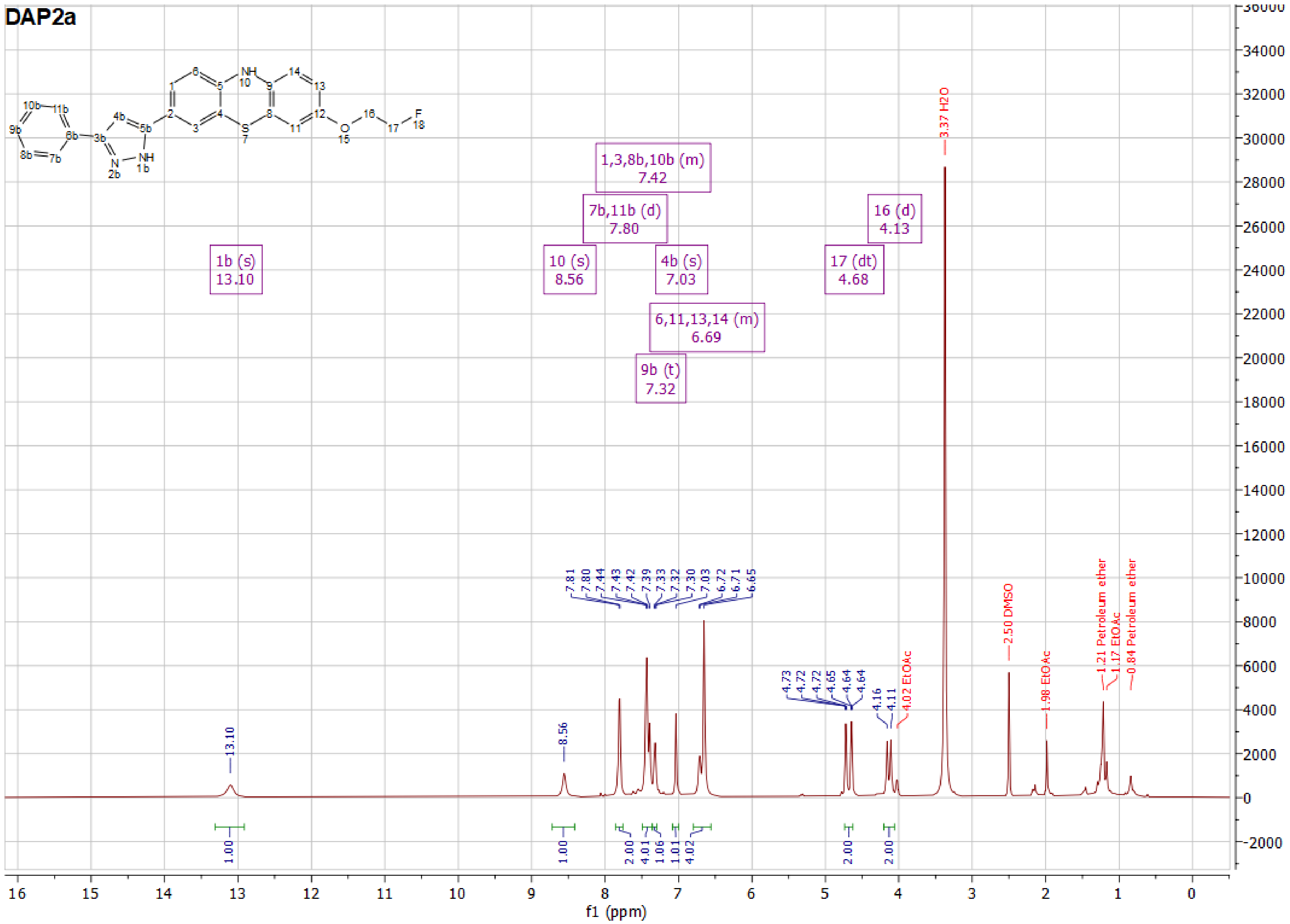
DAP2a
1b (s)
13.10
10 (s)
8.56
7b,11b (d)
7.80
1,3,8b,10b (m)
7.42
4b (s)
7.03
9b (t)
7.32
6,11,13,14 (m)
6.69
17 (dt)
4.68
16 (d)
4.13
3.37 H2O
2.50 DMSO
4.02 EtOAc
1.98 EtOAc
1.21 Petroleum ether
1.17 EtOAc
0.84 Petroleum ether
f1 (ppm)

6

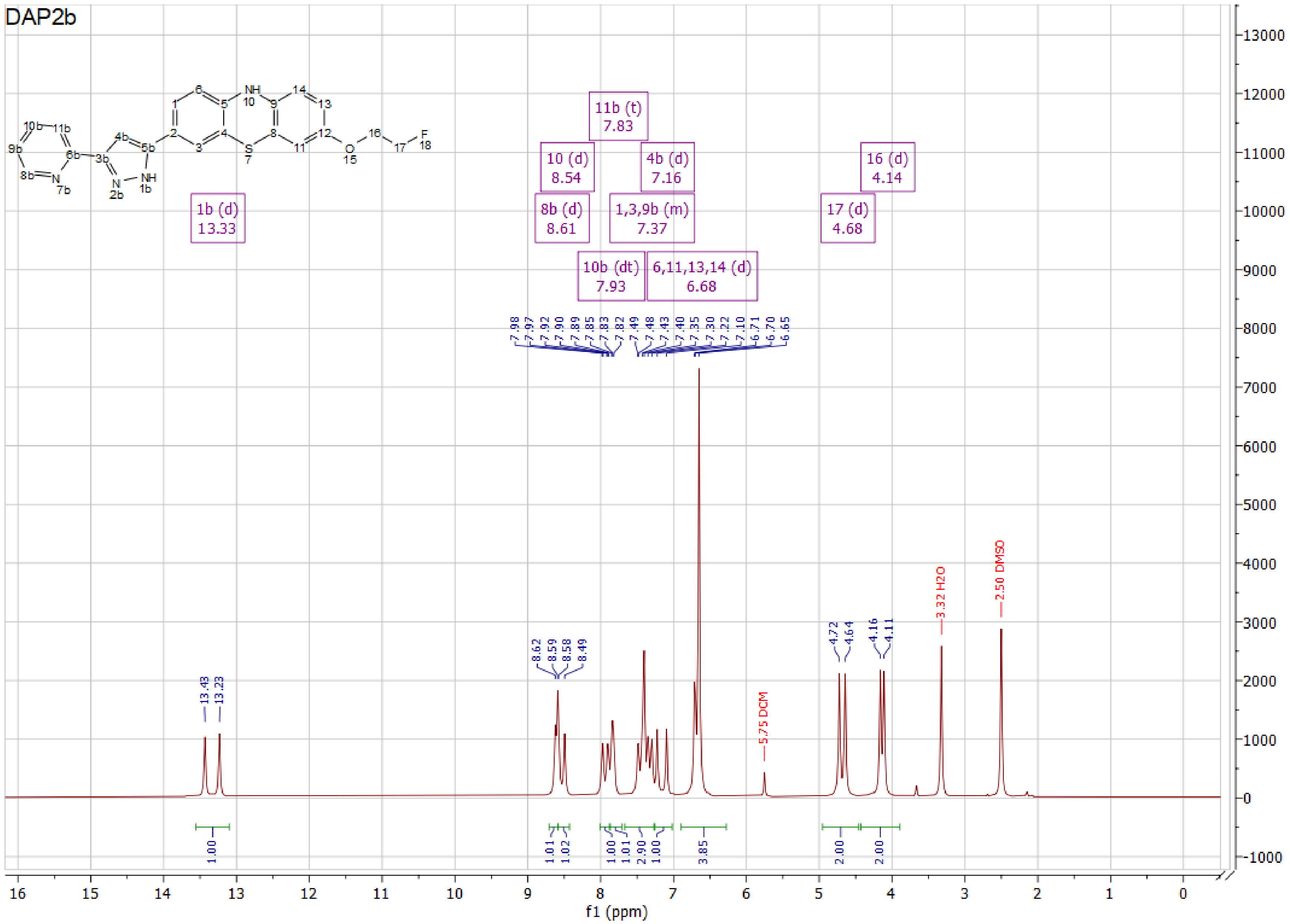
DAP2b
1b (d)
13.33
10 (d)
8.54
8b (d)
8.61
11b (t)
7.83
4b (d)
7.16
1,3,9b (m)
7.37
10b (dt)
7.93
6,11,13,14 (d)
6.68
16 (d)
4.14
17 (d)
4.68
5.75 DCM
3.32 H2O
2.50 DMSO
f1 (ppm)

7

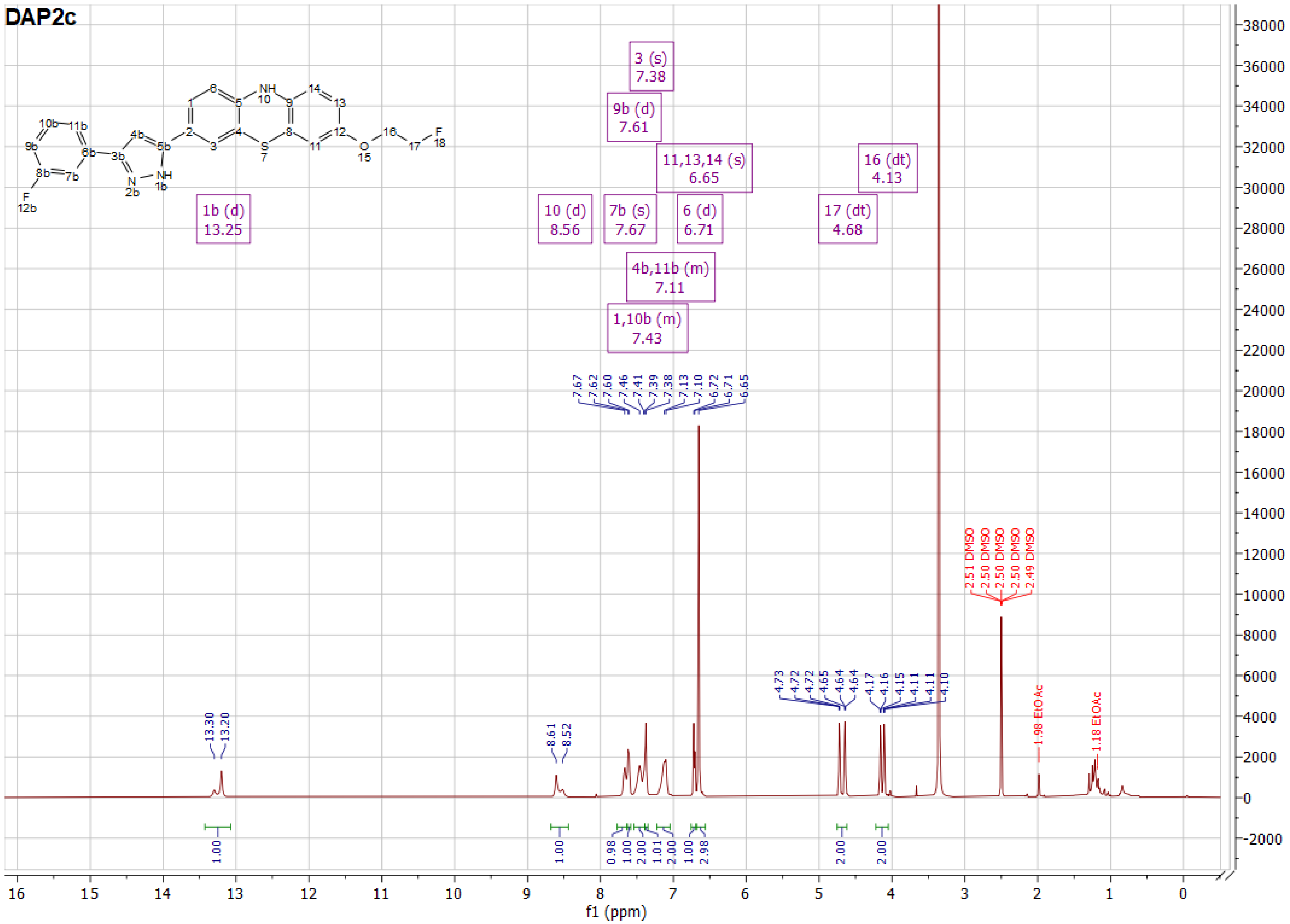

DAP2c
3 (s)
7.38
9b (d)
7.61
11,13,14 (s)
6.65
16 (dt)
4.13
1b (d)
13.25
10 (d)
8.56
7b (s)
7.67
6 (d)
6.71
17 (dt)
4.68
4b,11b (m)
7.11
1,10b (m)
7.43
2.51 DMSO
2.50 DMSO
2.50 DMSO
2.50 DMSO
2.49 DMSO
1.98 EtOAc
1.18 EtOAc
f1 (ppm)

8

9

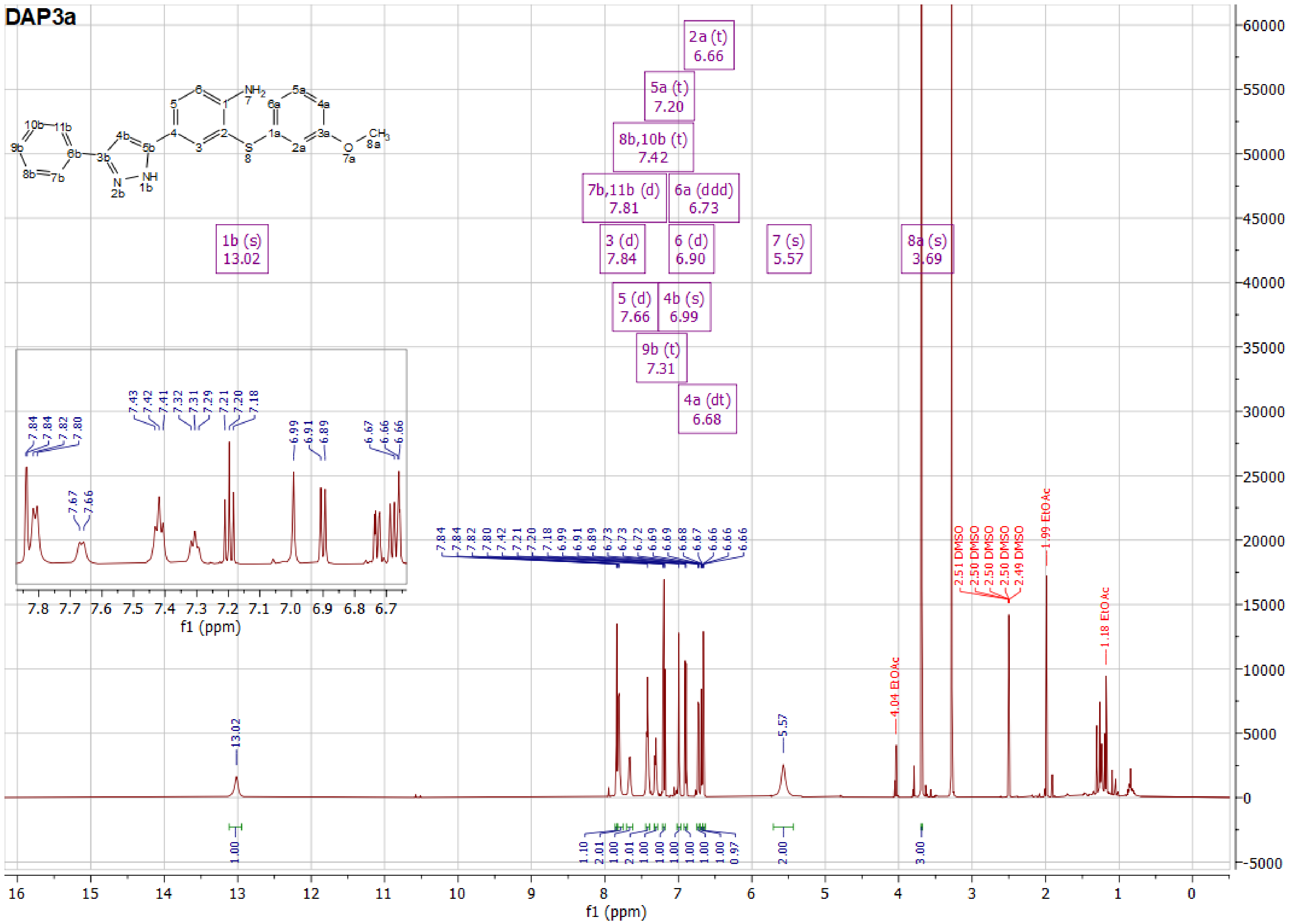
DAP3a
2a (t) 6.66
5a (t) 7.20
8b,10b (t) 7.42
7b,11b (d) 7.81
6a (ddd) 6.73
1b (s) 13.02
3 (d) 7.84
6 (d) 6.90
7 (s) 5.57
8a (s) 3.69
5 (d) 7.66
4b (s) 6.99
9b (t) 7.31
4a (dt) 6.68
4.04 EtOAc
2.51 DMSO
2.50 DMSO
2.50 DMSO
2.50 DMSO
2.49 DMSO
1.99 EtOAc
1.18 EtOAc
f1 (ppm)

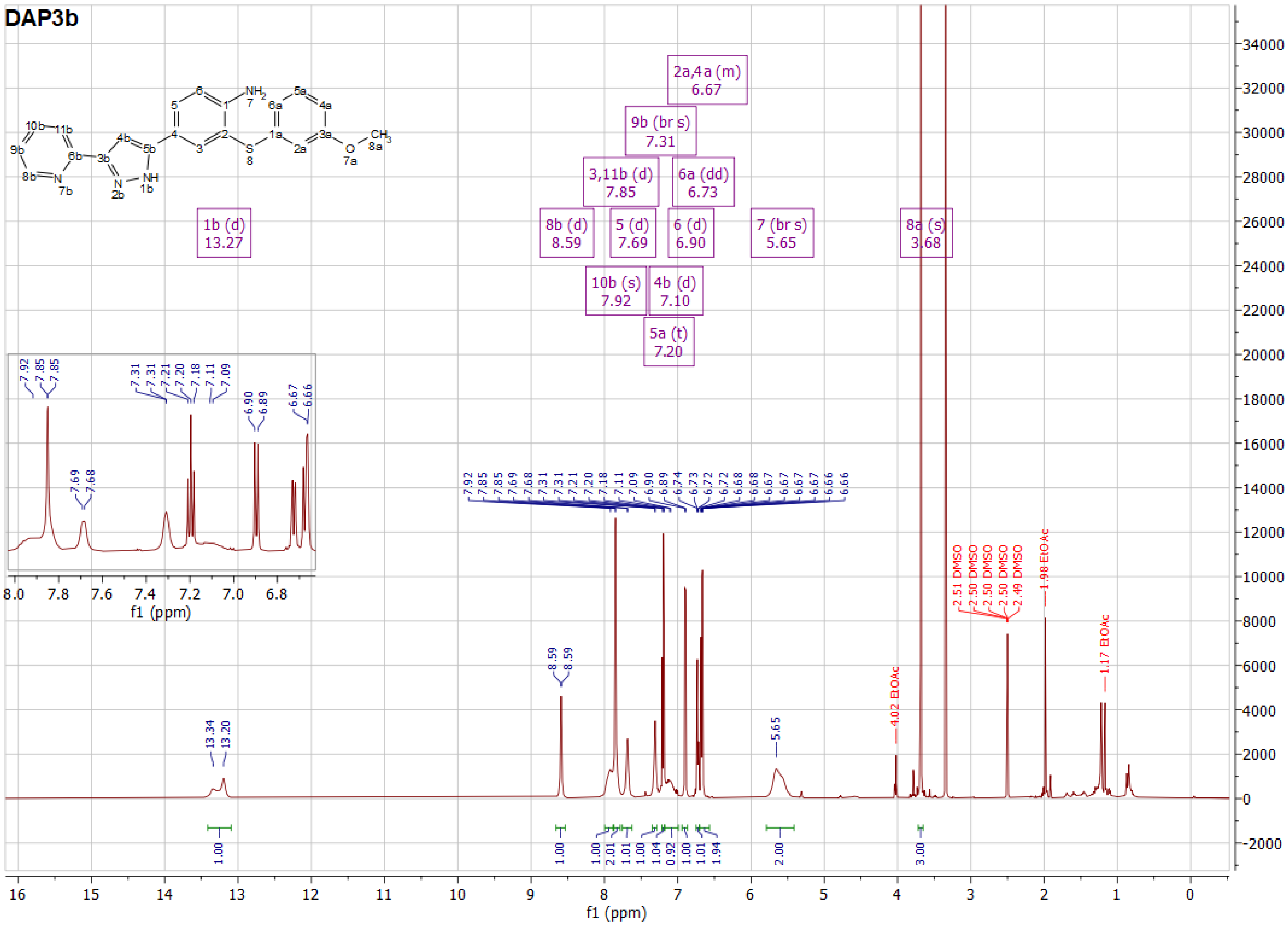
DAP3b
1b (d) 13.27
8b (d) 8.59
3,11b (d) 7.85
10b (s) 7.92
5 (d) 7.69
9b (br s) 7.31
5a (t) 7.20
4b (d) 7.10
6 (d) 6.90
6a (dd) 6.73
2a,4a (m) 6.67
7 (br s) 5.65
8a (s) 3.68
f1 (ppm)

10

11

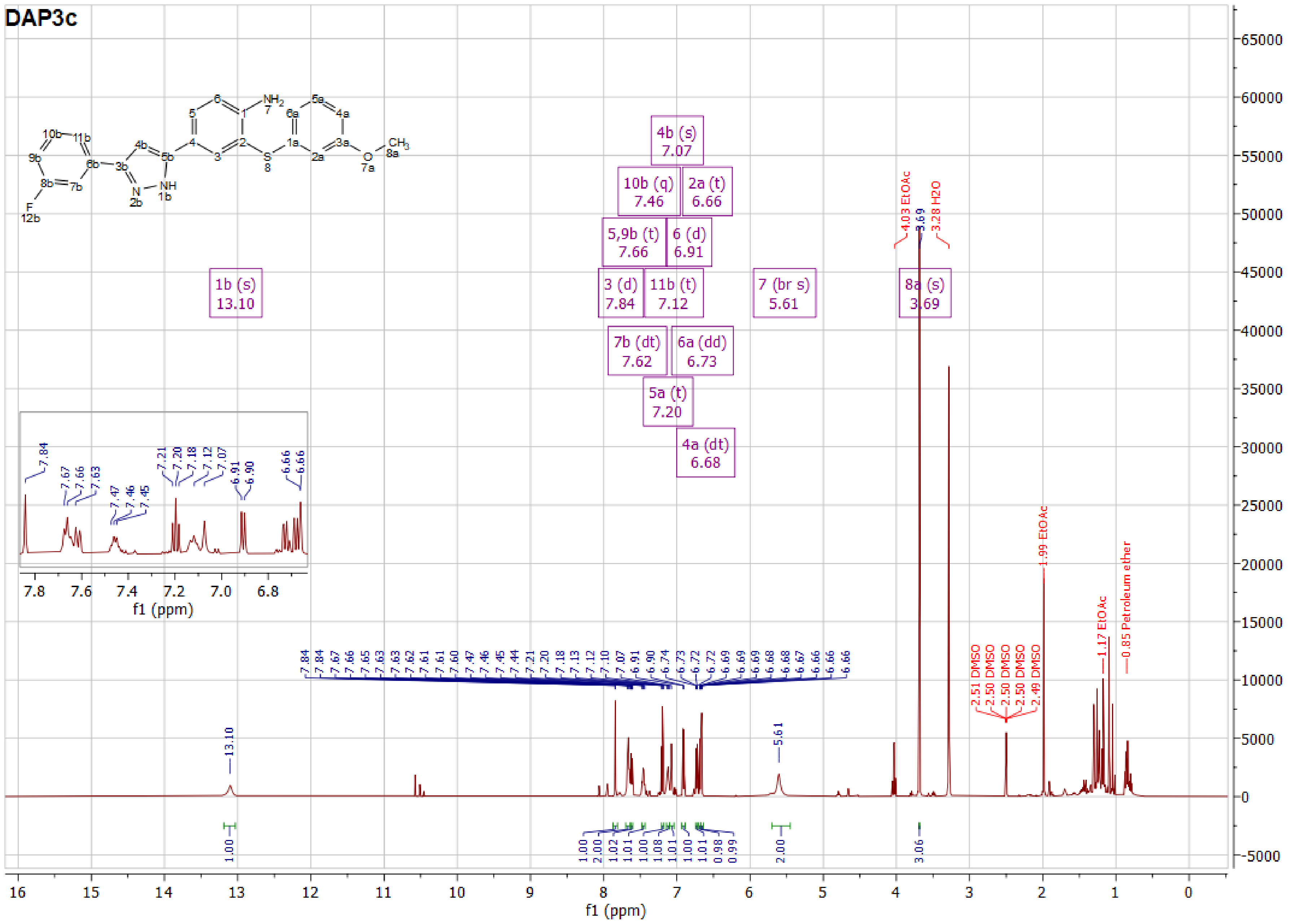
DAP3c
1b (s)
13.10
4b (s)
7.07
10b (q)
7.46
2a (t)
6.66
5,9b (t)
7.66
6 (d)
6.91
3 (d)
7.84
11b (t)
7.12
7 (br s)
5.61
8a (s)
3.69
7b (dt)
7.62
6a (dd)
6.73
5a (t)
7.20
4a (dt)
6.68
4.03 EtOAc
3.69
3.28 H2O
1.99 EtOAc
1.17 EtOAc
0.85 Petroleum ether
2.51 DMSO
2.50 DMSO
2.50 DMSO
2.50 DMSO
2.49 DMSO
f1 (ppm)
